# Attenuated SIRT1 Activity Leads to PER2 Cytoplasmic Localization and Dampens the Amplitude of *Bmal1* Promoter-Driven Circadian Oscillation

**DOI:** 10.3389/fnins.2021.647589

**Published:** 2021-05-24

**Authors:** Atsushige Ashimori, Yasukazu Nakahata, Toshiya Sato, Yuichiro Fukamizu, Takaaki Matsui, Hikari Yoshitane, Yoshitaka Fukada, Kazuyuki Shinohara, Yasumasa Bessho

**Affiliations:** ^1^Laboratory of Gene Regulation Research, Division of Biological Science, Graduate School of Science and Technology, Nara Institute of Science and Technology, Nara, Japan; ^2^Department of Neurobiology and Behavior, Graduate School of Biomedical Sciences, Nagasaki University, Nagasaki, Japan; ^3^Department of Ophthalmology, Graduate School of Medicine, Yamaguchi University, Yamaguchi, Japan; ^4^Research and Development Division, Mitsubishi Corporation Life Sciences Limited, Tokyo, Japan; ^5^Department of Biological Sciences, School of Science, The University of Tokyo, Tokyo, Japan

**Keywords:** circadian clock, PER2, NAD^+^, SIRT1, subcelluar localization

## Abstract

The circadian clock possesses robust systems to maintain the rhythm approximately 24 h, from cellular to organismal levels, whereas aging is known to be one of the risk factors linked to the alternation of circadian physiology and behavior. The amount of many metabolites in the cells/body is altered with the aging process, and the most prominent metabolite among them is the oxidized form of nicotinamide adenine dinucleotide (NAD^+^), which is associated with posttranslational modifications of acetylation and poly-ADP-ribosylation status of circadian clock proteins and decreases with aging. However, how low NAD^+^ condition in cells, which mimics aged or pathophysiological conditions, affects the circadian clock is largely unknown. Here, we show that low NAD^+^ in cultured cells promotes PER2 to be retained in the cytoplasm through the NAD^+^/SIRT1 axis, which leads to the attenuated amplitude of *Bmal1* promoter-driven luciferase oscillation. We found that, among the core clock proteins, PER2 is mainly affected in its subcellular localization by NAD^+^ amount, and a higher cytoplasmic PER2 localization was observed under low NAD^+^ condition. We further found that NAD^+^-dependent deacetylase SIRT1 is the regulator of PER2 subcellular localization. Thus, we anticipate that the altered PER2 subcellular localization by low NAD^+^ is one of the complex changes that occurs in the aged circadian clock.

## Introduction

The oxidized form of nicotinamide adenine dinucleotide (NAD^+^) found in all living cells plays critical roles in a wide range of physiological processes. NAD^+^ acts as a coenzyme for enzymes which are involved in energy metabolism and homeostasis pathways such as glycolysis, TCA cycle, and oxidative phosphorylation (Campbell, 1995). NAD^+^ also acts as a co-substrate for enzymes such as sirtuins (SIRTs) and poly(ADP-ribose) polymerases (PARPs) to regulate a wide array of cellular processes such as survival/cell death, circadian clock, and aging (Imai and Guarente, 2014; Verdin, 2015). In mammals, cellular NAD^+^ is primarily biosynthesized by the NAD^+^ salvage pathway, in which nicotinamide phosphoribosyltransferase (NAMPT) is the rate-limiting enzyme and converts nicotinamide into nicotinamide mononucleotide (NMN), the NAD^+^ precursor (Revollo et al., 2007; Imai and Guarente, 2014; Nakahata and Bessho, 2016).

NAD^+^ amount declines during the aging process, causing defects in nuclear and mitochondrial functions and resulting in many age-associated diseases (Gomes et al., 2013; Mouchiroud et al., 2013). Intriguingly, administration of NAD^+^ precursors such as NMN and nicotinamide riboside (NR) can ameliorate many age-associated pathologies, thereby leading to healthy longevity (Yoshino et al., 2018; Custodero et al., 2020; Hong et al., 2020). Similar to organismal aging, NAD^+^ amount is also reported to decline with cellular aging, i.e., cellular senescence (Khaidizar et al., 2017). Overexpression of NAMPT in primary fibroblast cells delays the onset of replicative senescence (van der Veer et al., 2007; Khaidizar et al., 2017) and confers a protective effect against stress-induced premature senescence (Nuriliani et al., 2020). These reports demonstrate that a decline in NAD^+^ is one of the causes to induce cellular senescence as well as organismal aging.

NAD^+^ in cells and organs demonstrates circadian oscillation due to the circadian clock regulation of *Nampt* gene expression (Nakahata et al., 2009; Ramsey et al., 2009). Moreover, NAD^+^ is associated with the transcriptional regulation of circadian clock genes through the regulation of SIRT1, SIRT6, and PARP1 activities (Asher et al., 2008, 2010; Nakahata et al., 2008; Masri et al., 2014). These findings demonstrate that circadian clock and NAD^+^ metabolism are mutually regulated (Imai, 2010; Nakahata and Bessho, 2016). Since NAD^+^ amount declines with senescence/aging, circadian clock properties are expected to be altered by senescence/aging. Indeed, circadian clocks in primary cells, tissues, and animals show altered circadian properties by senescence/aging. The circadian amplitude at the transcriptional (Kunieda et al., 2006; Nakamura et al., 2015; Ahmed et al., 2019), neural activity (Nakamura et al., 2011), and locomotor activity levels (Pittendrigh and Daan, 1974; Witting et al., 1994; Valentinuzzi et al., 1997; Sellix et al., 2012) declines with aging. The period of circadian genes alters with senescence/aging in primary human fibroblasts and *ex vivo* mouse SCN slices (Nakamura et al., 2015; Ahmed et al., 2019), although it is still controversial at the organismal level (Valentinuzzi et al., 1997; Aujard et al., 2001; Kolker et al., 2004). However, only a few researches have looked into the molecular mechanisms of how the circadian clock is affected by senescence/aging (Chang and Guarente, 2014; Zwighaft et al., 2015; Levine et al., 2020).

We have reported that a decline in NAD^+^ keeps activating the transcription of E-box-regulated circadian clock genes *via* hyperacetylation of lysine 9/14 residues on histone H3, due to the inactivation of NAD^+^-dependent deacetylase, SIRT1 (Nakahata et al., 2008). In addition to that report, some investigators have also demonstrated that NAD^+^ regulates other circadian clock components: SIRT1 deacetylates PER2, resulting in its stabilization (Asher et al., 2008); SIRT1 deacetylates PGC1α to activate *Bmal1* transcription (Chang and Guarente, 2014); PARP-1, an NAD^+^-dependent ADP-ribosyltransferase, poly(ADP-ribosyl)ates CLOCK (Asher et al., 2010); and the inhibitor of SIRT1 changes the ratio of PER2 subcellular localization (Miki et al., 2012).

The aforementioned findings prompted us to reveal the molecular mechanisms of how NAD^+^ amount, especially low NAD^+^, affects the circadian clock system, which might give some hints to unravel molecular links between aging and circadian clock. In this study, we reduced intracellular NAD^+^ amount by inhibiting NAMPT enzymatic activity and found that it attenuates the amplitude of *Bmal1* promoter-driven luciferase oscillation and promotes cytoplasmic localization of PER2, leading to attenuated CRY/PER-dependent repression, thereby enhancing E-box-regulated circadian gene expressions. At the molecular level, we revealed that the translocation of PER2 with low NAD^+^ is dependent upon SIRT1 activity, but not PARP1 activity.

## Materials and Methods

### Reagents, Antibodies, and Plasmids

FK866, GMX1778, nicotinamide, EX-527, and PJ34 were purchased from the Axon Medchem, AdipoGen Life Sciences, Sigma, Cayman Chemical, and Abcam, respectively. Nicotinamide mononucleotide (NMN) was provided by the Mitsubishi Corporation Life Sciences Limited, Japan. Anti-Per2 (PM083), anti-Lamin B1 (PM064), anti-Myc-tag (M047-3), and anti-DDDDK-tag (M185-3) were purchased from the MBL, Japan. Anti-α-tubulin (T6074) and the secondary anti-mouse antibody Alexa Fluor Plus 488 (A32723) were purchased from the Sigma-Aldrich and Thermo Fisher Scientific, respectively. PAR/pADPr antibody was purchased from the R&D Systems. Anti-Bmal1 (ab93806) and anti-acetyl Bmal1 (Lys538) were purchased from the Abcam and Merck, respectively. Secondary anti-mouse (NA931V) or rabbit (NA934) antibodies conjugated with HRP were purchased from the GE Healthcare. Myc-mClock/pcDNA3 was subcloned from Myc-mClock/pSG5 as described (Doi et al., 2006). 5xMyc-mCry1-Flag/pCS2 and Myc-mBmal1/pcDNA3 were described as previously (Hirayama et al., 2007). Flag-hRORα1/pcDNA3, Flag-hRORγ/pcDNA3, Flag-mRev-erbα/pcDNA3, and Flag-mRev-erbβ/pcDNA3 were inserted a single Flag epitope into hRORα1/pcDNA3, hRORγ/pcDNA3, mRev-erbα/pcDNA3, and mRev-erbβ/pcDNA3, respectively, provided by Akashi and Takumi (2005). Flag-mCry1/pcDNA3, 2xMyc-Per2-NLS/pcDNA3, and 2xMyc-Per2-NES/pcDNA3 were kind gifts of Akashi et al. (2014). pLLX-shRNA and pLLX-scrambled shRNA were kindly provided by ME Greenberg (Zhou et al., 2006).

### Cell Culture

NIH3T3 cells were cultured in DMEM–4.5 g/L glucose (Nacalai Tesque, Japan) supplemented with 10% FBS (Sigma) and antibiotics (100 units/ml penicillin, 100 μg/ml streptomycin, Nacalai Tesque, Japan) at 37°C and 5% CO_2_ in a humidified incubator. Cells were treated with FK866/GMX1778 with or without 1 mM nicotinamide mononucleotide for 24 h and harvested for measuring NAD^+^ amount, performing qPCR, immunofluorescence, and western blotting. For experiments analyzing circadian oscillations of clock genes, luciferase, and PER2 protein, cells were pretreated with FK866 for 24 h before Dex synchronization and also treated with FK866 after synchronization.

### Quantification of Intracellular NAD^+^ by HPLC

NAD^+^ was measured by an HPLC system, an Agilent 1260 Infinity Binary LC System with guard (Polaris C18-A, MetaGuard, 5 μm, 4.6 mm; A2000MG, Agilent) and analytical (Polaris 5 C18-A 4.6 × 150 mm; A2000150 × 046, Agilent) columns, following the protocol reported previously (Yoshino and Imai, 2013; Khaidizar et al., 2017).

### SDS-PAGE, Western Blotting Analysis, RNA Extraction, and qPCR

Protocols for SDS-PAGE, western blotting analysis, RNA extraction, and qPCR were followed as described previously (Khaidizar et al., 2017). Protein measurement was performed using 660 Protein Assay Reagent (Thermo Fisher Scientific) according to the manufacturer’s instruction. The sequences for qPCR primers are shown in Supplementary Table 1.

### Real-Time Luciferase Monitoring Assay

The method was described previously (Ahmed et al., 2019).

### Immunofluorescence

NIH3T3 cells were seeded on a sterilized cover glass (Matsunami; 24 × 24 mm; thickness no. 1) placed on a six-well plate so as to have 4 × 10^5^ cells/well as a single experiment. Cells were co-transfected with indicated expression vectors using FuGENE HD (Promega) and cultured for 48 h. The cells after washing twice with phosphate buffered saline (PBS) were fixed with 1 ml of 4% paraformaldehyde for 15 min with shaking at room temperature. Then, cells were washed three times with PBS and permeabilized with 1 ml of 0.5% Triton X-100 for 10 min with shaking at room temperature. After blocking the cells with 1% bovine serum albumin (BSA) for 30 min at room temperature, the cells were washed three times with PBS and incubated with Myc antibody diluted with 1% BSA/PBS for 1 h at room temperature. Then, cells were incubated with secondary antibody conjugated with Alexa Fluor Plus 488 diluted with 1% BSA/PBS for 1 h at room temperature. Nuclei were also stained with 1 μg/ml Hoechst 33342 (Nacalai Tesque, Japan) to justify subcellular localizations of clock proteins. Fluorescent images were acquired with a confocal laser scanning microscope (ZEISS; LSM 700), and subcellular localizations of clock proteins were counted at least 100 cells. We interpreted the result for nuclear localization of PER2/CRY1 (N) as positive when fluorescence intensity of PER2/CRY1 signal that overlapped with Hoechst 33342 signal was higher than the fluorescence intensity of PER2/CRY1 in the cytoplasm (Supplementary Figure 1). Meanwhile, we interpreted the result for cytoplasmic localization of PER2/CRY1 (C) as positive when PER2/CRY1 signal that did not overlap with Hoechst 33342 signal exhibited higher fluorescence intensity than PER2/CRY1 signal intensity that overlapped with Hoechst 33342 signal. Finally, we interpreted the result for nuclear and cytoplasm localization of PER2/CRY1 (N + C) as positive when PER2/CRY1 fluorescence signal at both nucleus and cytoplasm had the same intensities. We performed the experiments independently for at least three times and showed the data as mean ± SEM.

### Nuclear Extract From NIH3T3 Cells

NIH3T3 cells treated with or without 7.5 nM FK866 for 24 h in a 10-cm dish were synchronized with 100 μM dexamethasone for 1 h. Synchronized cells after 24–44 h were collected every 4 h. Cells were washed twice with PBS and harvested by cell scrapers, centrifuged at 300 × *g* for 5 min at 4°C, and resuspended in 50 μl lysis buffer [10 mM HEPES (pH 7.9), 1.5 mM MgCl_2_, 10 mM KCl]. Cells were vortexed for 10 s and allowed to stand on ice for 10 min, and the same step was repeated again to extract cell components other than the nuclear fraction. The nuclear fraction was washed three times with lysis buffer, added with 50 μl RIPA buffer [50 mM Tris–HCl (pH 7.4), 150 mM NaCl_2_, 1 mM EDTA, 0.2% SDS, 1% NP-40], vortexed for 30 s, and then allowed to stand on ice for 20 min. The nuclear fraction was centrifuged at 15,000 × *g* for 5 min at 4°C, and the supernatant was collected as a nuclear extract. Total cell extract was obtained by extracting cells with RIPA buffer.

### Dual-Luciferase Promoter Assay

NIH3T3 cells seeded in a 24-well plate were co-transfected with 500 ng of *Per1* firefly reporter, 50 ng of *EF1a Renilla* reporter, and indicated expression vectors using FuGENE HD (Roche). Samples were prepared using a Dual-Luciferase promoter assay kit (Promega) according to their instructions and analyzed by a multiwell luminescence measurement plate reader (Mithras, LB940). Firefly luciferase activity of the reporter was normalized by the activity of *Renilla* luciferase under control of EF1α promoter.

### Establishment of Sirt1 Knockdown Cell

To establish Sirt1 knockdown cells, lentivirus vector pLLX-shRNA (Zhou et al., 2006), which expresses GFP and a puromycin resistant gene, was used. After virus infection to *Bmal1*-luc/NIH3T3 (Yoshitane et al., 2012), cells were selected by puromycin for 1 week and confirmed by GFP fluorescence (all cells were ∼100% GFP positive). Three different target sequences for mouse *Sirt1* and scramble sequence are shown in Supplementary Table 2.

### Statistics

Values are reported as mean ± SEM. Statistical differences were determined by a Student’s two-tailed *t*-test. Statistical significance is displayed as ^∗^*p* < 0.05, ^∗∗^*p* < 0.01, or ^∗∗∗^*p* < 0.001.

## Results

**FIGURE 1:**
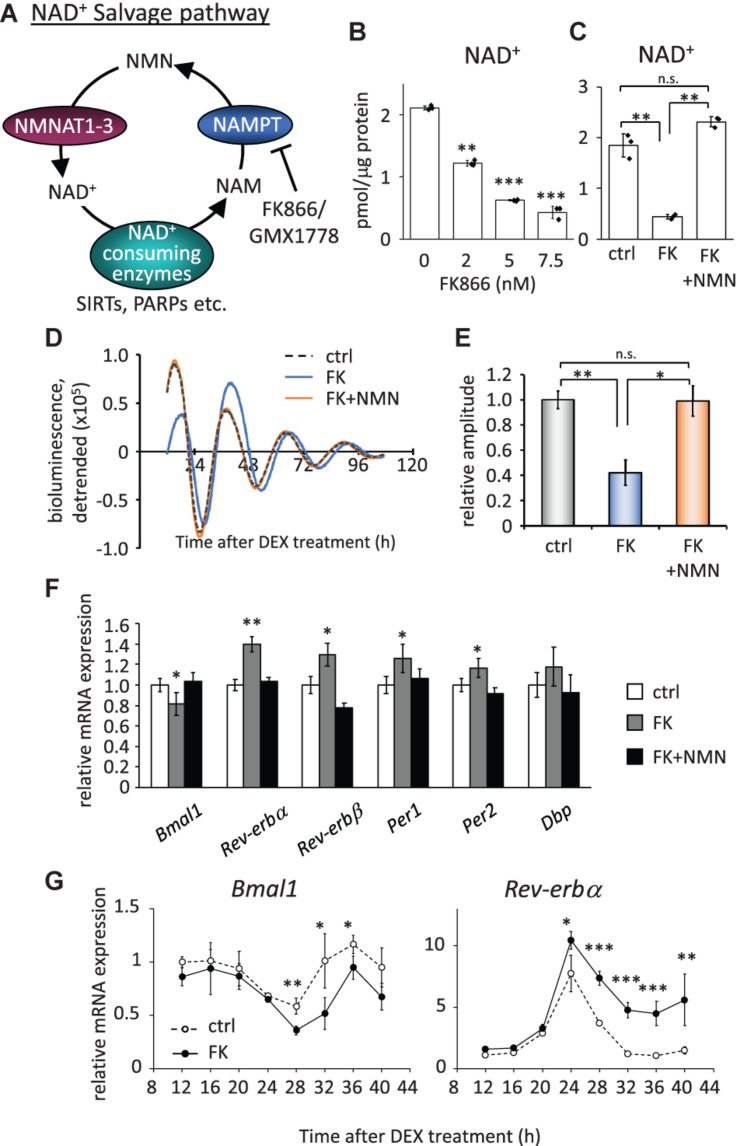
FK866 changes the expression levels of circadian genes, which is abrogated by co-treatment with NMN. **(A)** Scheme of the NAD^+^ salvage pathway. NAM, nicotinamide; NMN, nicotinamide mononucleotide; NAMPT, nicotinamide phosphoribosyltransferase; NMNAT1-3, nicotinamide mononucleotide adenylyltransferase 1-3. **(B)** NAD^+^ amount after 24 h treatment of FK866 at indicated concentrations was measured by HPLC. NAD^+^ amount was normalized by protein amount. **(C)** NAD^+^ amount after 24 h treatment of 7.5 nM FK866 with or without 1 mM NMN was measured by HPLC. NAD^+^ amount was normalized by protein amount. **(D)** *Bmal1*-luc oscillation patterns in NIH3T3 cells treated with FK866 with or without NMN were monitored by using a real-time luciferase monitoring system. One representative result is shown for each condition. **(E)** Relative amplitudes were analyzed. In this study, the amplitude was defined as the height from the first peak to the first trough. The value of control (ctrl) was set to 1. **(F)** Circadian gene expression levels after treatment with 7.5 nM FK866 alone or 7.5 nM FK866 and 1 mM NMN were quantified by qPCR. Each sample was normalized by the amount of 18S rRNA. Each gene expression level in untreated cells (ctrl) was set to 1. **(G)** Circadian profiles of *Bmal1* and *Rev-erb*α synchronized by DEX were quantified. Each sample was normalized by 18S rRNA. Time 12 of control cells was set to 1 for each gene. All data represented here, except **(E)** (*n* = 4), are the mean ± SEM of three independent samples. ^∗^*p* < 0.05, ^∗∗^*p* < 0.01, and ^∗∗∗^*p* < 0.001, compared to each of the untreated control by Student’s two-tailed *t-*test.

To investigate how the circadian clock system is affected by low NAD^+^ in cells, we first evaluated how much NAD^+^ was decreased by the inhibition of the rate-limiting enzyme, NAMPT, in the NAD^+^ salvage pathway (Figure 1A). We used FK866 (Hasmann and Schemainda, 2003) to inhibit the NAMPT activity and found that 2.0, 5.0, and 7.5 nM FK866 treatment decreased NAD^+^ to approximately 50, 33, and 25%, respectively (Figure 1B). In addition, the acetylated form of BMAL1, which has been reported to be increased by FK866 treatment (Nakahata et al., 2009), was increased by FK866 treatment in a dose-dependent manner (Supplementary Figure 2A). We further confirmed that the effect of FK866 treatment at 7.5 nM on NAD^+^ amount was abrogated by the co-treatment with the NAD^+^ precursor, NMN (Figure 1C). A similar tendency was observed by another NAMPT inhibitor, GMX1778 (Watson et al., 2009) (Supplementary Figures 3A,B). We then performed a real-time luciferase assay to address whether circadian clock properties are affected by low NAD^+^. To address that, we used NIH3T3 cells stably expressing *Bmal1* promoter-driven *luciferase* (Yoshitane et al., 2012). The amplitude, which was defined in this study as the height from the first peak to the first trough, of *Bmal1* promoter-driven luciferase oscillation was attenuated in FK866-treated cells, compared with that in control cells (Figures 1D,E). A delayed phase was also observed in FK866-treated *Bmal1*-luc oscillation. Intriguingly, the attenuated amplitude and delayed phase were abrogated in FK866 and NMN co-treated cells, indicating that the amplitude and phase are regulated by NAD^+^ amount. The reason why these alterations were abrogated after 60 h Dex treatment is presumably due to the recovery of intracellular NAD^+^ amount (Supplementary Figure 4). We further checked endogenous circadian gene expressions in unsynchronized cells with low NAD^+^. Among core circadian genes, E-box-regulated genes, such as *Rev-erb*α, *Rev-erb*β, *Per1*, and *Per2*, were upregulated; however, the RORE-regulated gene, *Bmal1*, was downregulated (Figure 1F). Moreover, these alterations were abrogated in FK866 and NMN co-treated cells, indicating that the alterations of these genes are regulated by NAD^+^ amount. Furthermore, we demonstrated that circadian profiles of endogenous *Bmal1* and *Rev-erb*α transcripts were also affected, lower *Bmal1* and higher *Rev-erb*α, in synchronized cells with low NAD^+^ (Figure 1G). These results demonstrate that low NAD^+^ alters transcript levels of not only E-box-regulated genes as we have reported previously (Nakahata et al., 2008), but also RORE-regulated genes, which presumably regulate the amplitude of circadian clocks.

**FIGURE 2:**
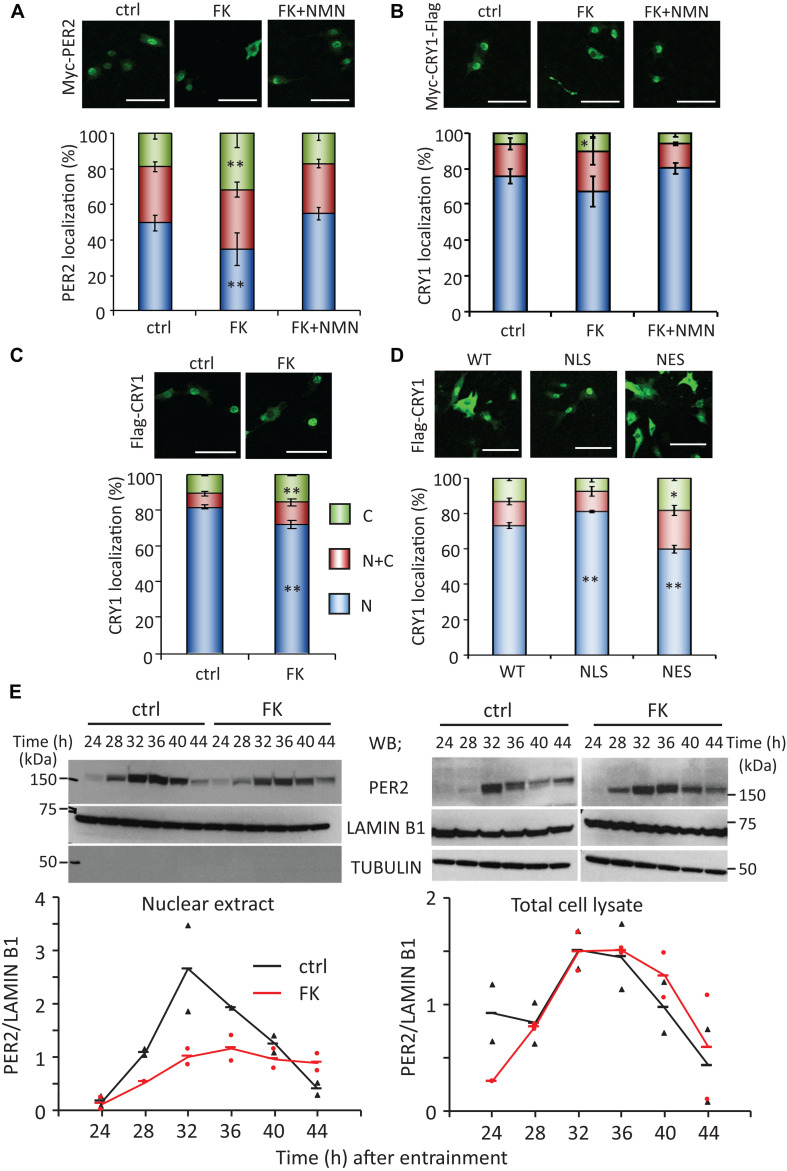
FK866 changes PER2 subcellular localization, which is abrogated by co-treatment with NMN. [**(A–D)**, top] NIH3T3 cells expressing Myc-PER2 **(A)**, Myc-CRY1-Flag **(B)**, or Myc-PER2/Flag-CRY1 **(C)** were immunostained with antibody to Myc or Flag (top). Cells were either untreated (ctrl) or treated with 7.5 nM FK866 (FK) or 7.5 nM FK866 and 1 mM NMN (FK + NMN). NIH3T3 cells expressing Flag-CRY1 and either Myc-PER2-WT, Myc-PER2-NLS, or Myc-PER2-NES were immunostained with antibody to Flag **(D)**. The representative images were captured by a confocal laser scanning microscope. White scale bars represent 100 μm. [**(A–D)**, bottom] Subcellular localizations were counted and quantified; N, nucleus; C, cytoplasm; N + C, both nucleus and cytoplasm. The data represented are the mean ± SEM of at least three independent samples. ^∗^*p* < 0.05, ^∗∗^*p* < 0.01, compared to each subcellular localization in control cells by Student’s two-tailed *t-*test. **(E)** PER2 protein levels in nuclear extract and total cell lysate were detected under the 7.5 nM FK866-treated condition (top). PER2 protein levels were quantified using ImageJ software (bottom). Each sample was normalized by LAMIN B1 protein level. TUBULIN was used to confirm fractionation. The data represented as filled circle and triangle are individual data of two independent samples and bars are the average of two samples.

As our previous studies have demonstrated that the circadian gene expressions are epigenetically regulated by NAD^+^-dependent deacetylases, SIRT1 and SIRT6 (Nakahata et al., 2008; Masri et al., 2014), in this study, we sought to find out whether the properties of circadian clock proteins, such as protein stability and subcellular localization, are regulated by NAD^+^ amount. First, whether low NAD^+^ affects protein stability of clock proteins was investigated using cycloheximide, which is an inhibitor of protein biosynthesis due to its prevention in translational elongation. However, the stabilities of CLOCK, BMAL1, PER2, and CRY1 were not significantly affected by low NAD^+^ (Supplementary Figure 5). Next, whether low NAD^+^ affects subcellular localization of clock proteins was investigated (Figures 2A,B and Supplementary Figure 6). Among clock proteins tested in this study, subcellular localization of PER2 was clearly affected by FK866 treatment (Figure 2A). Cytoplasmic and nuclear PER2 under control condition were 18.8 ± 3.4 and 49.3 ± 4.2%, respectively, whereas cytoplasmic and nuclear PER2 under FK866-treated condition were 32.0 ± 7.9% (*p* = 5.1 × 10^–3^, compared with cytoplasmic PER2 under control condition) and 34.5 ± 9.2% (*p* = 3.8 × 10^–3^, compared with nuclear PER2 under control condition), respectively. Furthermore, GMX1778 treatment also changed the ratio of PER2 subcellular localization from 22.2 ± 0.5 to 26.3 ± 0.1% in the cytoplasm (*p* = 1.0 × 10^–4^) and from 62.0 ± 1.9 to 50.0 ± 0.6% in the nucleus (*p* = 2.0 × 10^–4^) (Supplementary Figure 7). Importantly, the effect of FK866 or GMX1778 on PER2 subcellular localization was abrogated when cells were treated with both FK866 and NMN or GMX1778 and NMN (Figure 2A and Supplementary Figure 7). These results indicate that PER2 subcellular localizations are controlled by intracellular NAD^+^ amount; lower NAD^+^ promotes the translocation of PER2 from the nucleus to the cytoplasm. Similarly, cytoplasmic and nuclear CRY1 under control condition were 6.3 ± 0.9 and 75.6 ± 3.9%, respectively, while cytoplasmic and nuclear CRY1 under FK866-treated condition were 10.6 ± 2.8% (*p* = 0.026, compared with cytoplasmic CRY1 under control condition) and 67.1 ± 8.5% (*p* = 0.12, compared with nuclear CRY1 under control condition), respectively (Figure 2B), indicating the similarity to the change in PER2 by FK866 treatment. These results prompted us to investigate whether the increase in PER2 cytoplasmic localization triggered by FK866 induces the increase in CRY1 cytoplasmic localization, because it is well known that PER2 determines CRY1 localization (Albrecht et al., 2007). Cytoplasmic localizations of CRY1 co-expressing PER2 with or without FK866 were 15.4 ± 0.7 and 10.8 ± 0.8% (*p* = 4.8 × 10^–3^) and nuclear localizations were 71.8 ± 2.2 and 81.7 ± 1.3% (*p* = 8.6 × 10^–3^), respectively (Figure 2C). The alteration of CRY1 subcellular localization by FK866 treatment was more when PER2 was co-expressed, suggesting that the alteration of PER2 subcellular localization leads to that of CRY1.

To confirm whether the changes in PER2 subcellular localization alter CRY1 subcellular localizations, PER2 wild type (WT), C-terminal-tagged nuclear localization signal (-NLS), or C-terminal-tagged nuclear export signal (-NES) (Akashi et al., 2014) was co-expressed with CRY1. Similar to a previous report (Akashi et al., 2014), nuclear localizations of PER2-WT, PER2-NLS, and PER2-NES without CRY1 were 59.1 ± 2.1, 73.6 ± 1.3, and 37.0 ± 4.7%, respectively (Supplementary Figure 8A). As expected, subcellular localizations of CRY1 were followed by those of PER2. Compared with the ratio of nuclear CRY1 co-expressing with PER2-WT (73.1 ± 1.6%), the ratio of nuclear CRY1 was increased (81.1 ± 0.7%, *p* = 0.011) when PER2-NLS was co-expressed, whereas the ratio of nuclear CRY1 was decreased (59.8 ± 2.2%, *p* = 0.008) when PER2-NES was co-expressed (Figure 2D). These results are consistent with previous reports that PER2 determines CRY1 localization (Albrecht et al., 2007). Even when CRY1 was co-expressed with these PER2s, PER2 subcellular localizations were not affected (Supplementary Figure 8B). The results of Figures 2A–D suggest that localization of PER2 is directly affected by intracellular NAD^+^ amount and the intracellular localization of CRY1 follows that of PER2.

Since PER2 protein amount and subcellular localization show time-of-day variations, whether circadian subcellular localization of PER2 is affected by NAD^+^ amount was next investigated (Figure 2E). Although nuclear localization of PER2 clearly showed the time-of-day variation under the control condition as reported previously (Lee et al., 2001; Yagita et al., 2001), the nuclear accumulation of PER2 was reduced, but still showed circadian oscillation, under the FK866-treated condition (Figure 2E, left panel). Intriguingly, PER2 in total cell lysate under the FK866-treated condition was comparable with that under the control condition (Figure 2E, right panels), suggesting that the reduction of PER2 nuclear localization under the FK866-treated condition is not due to the reduction of PER2, but the decrease in PER2 nuclear entry and/or the increase in PER2 nuclear export.

**FIGURE 3:**
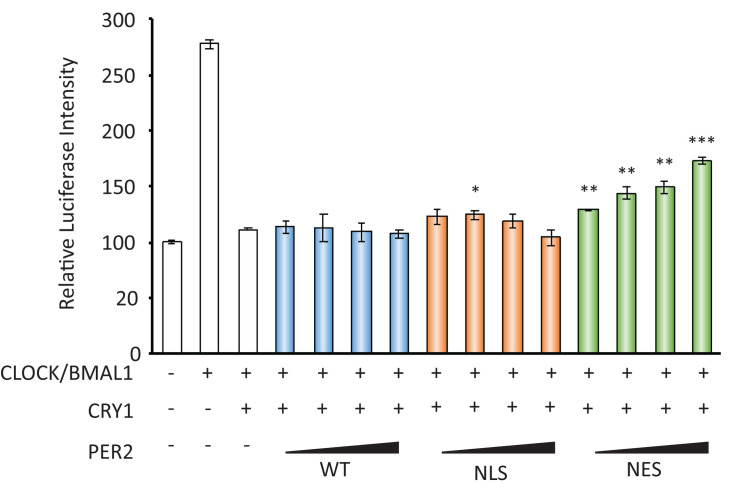
PER2 subcellular localization affects *Per1* gene expression. The effects of PER2-WT, PER2-NLS, and PER2-NES on per1 luciferase activity are shown; 100 ng of CLOCK, 100 ng of BMAL1, 10 ng of CRY1, 500 ng of *Per1* firefly reporter, and 50 ng of *EF1a Renilla* reporter were co-transfected in NIH3T3 cells. Indicated amounts of PER2-WT, PER2-NLS, or PER2-NES were also co-transfected in NIH3T3 cells. Firefly luciferase intensity was normalized by *Renilla* luciferase intensity. Basal *per1* promoter activity (far left bar) was set to 100. The data represented are the mean ± SEM of three independent samples. ^∗^*p* < 0.05, ^∗∗^*p* < 0.01, and ^∗∗∗^*p* < 0.001, compared with the sample with CLOCK/BMAL1 and CRY1, the third bar from left, by Student’s two-tailed *t-*test.

CRY acts as a transcriptional repressor with PER against CLOCK/BMAL1-dependent, namely E-box-regulated, transcriptions. As the decrease in nuclear localization of PER2 by FK866 suggested the attenuation of PER/CRY-dependent repression against E-box-regulated genes, we next addressed that possibility. To address that, luciferase reporter assay using *Per1* promoter that possesses several E-boxes was assessed using PER2-NES, which mimics PER2 subcellular localizations under the FK866-treated condition. As reported previously, *Per1* promoter activity was activated by CLOCK/BMAL1 and this activation was repressed by CRY1 (Figure 3). When PER2-NES was co-expressed, the repression by CRY1 against CLOCK/BMAL1-dependent *Per1* expression was attenuated; namely, the luciferase intensities were increased in a PER2-NES dose-dependent manner (Figure 3). On the other hand, when PER2-WT or PER2-NLS was co-expressed, the repression by CRY1 was not affected. The results from Figures 2E, 3 indicate that PER2 subcellular localization determines PER/CRY-dependent repression potential, and support that the mechanism of upregulation of E-box-regulated genes under the FK866-treated condition (Figure 1C) is due to the preferential cytoplasmic localization of PER2 with CRY1.

**FIGURE 4:**
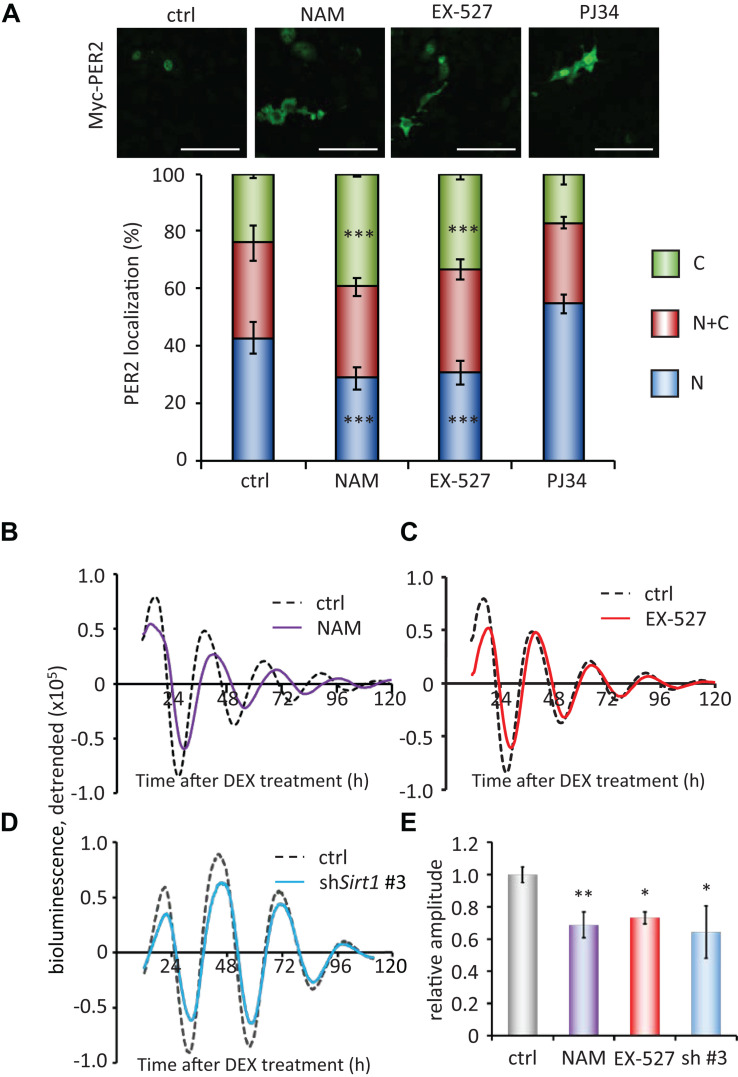
SIRT1 inhibitors, but not PARP1 inhibitor, regulate PER2 localization and attenuate the amplitude. **(A)** NIH3T3 cells expressing Myc-PER2 were immunostained with antibody to Myc. Cells were either untreated (ctrl), treated with 10 mM nicotinamide (NAM), 5 μM EX-527 or 10 μM PJ34 (top). The representative images were captured by a confocal laser scanning microscope. White scale bars represent 100 μm. (Bottom) Subcellular localizations were quantified; N, nucleus; C, cytoplasm; N + C, both nucleus and cytoplasm. The data represented are the mean ± SEM of independent three samples. ^∗∗∗^*p* < 0.001, compared to each subcellular localization in control (ctrl) cells by Student’s two-tailed *t* test. **(B,C)** *Bmal1*-luc oscillation patterns in NIH3T3 cells treated with either NAM or EX-527 were monitored by using a real-time luciferase monitoring system. One representative result is shown for each condition. **(D)** *Bmal1*-luc oscillation patterns in WT (ctrl) or *Sirt1* knockdown (sh*Sirt1* #3) NIH3T3 cells were monitored by using a real-time luciferase monitoring system. One representative result is shown for each condition. **(E)** Relative amplitudes were analyzed. The value of control (ctrl) was set to 1. The data represented are the mean ± SEM of independent 8, 5, 3 or 3 samples for the control, NAM, EX-627, or sh*Sirt1* #3, respectively. ^∗^*p* < 0.05, ^∗∗^*p* < 0.01, compared to the control (ctrl) by Student’s two-tailed *t-*test.

Finally, to investigate the molecular mechanisms of how PER2 subcellular localization is regulated, we pharmacologically inhibited NAD^+^-dependent enzymes, SIRT1 and PARP1. The SIRT1 inhibitors, 10 mM nicotinamide (NAM) or 5 μM EX-527 (Bitterman et al., 2002; Napper et al., 2005), significantly increased cytoplasmic PER2 from 24.1 ± 1.3 to 39.5 ± 0.5% (*p* = 4.0 × 10^–4^) and 33.5 ± 2.1% (*p* = 1.9 × 10^–4^), respectively, and decreased nuclear PER2 from 42.7 ± 5.5 to 28.7 ± 3.8% (*p* = 3.0 × 10^–4^) and 30.5 ± 4.2% (*p* = 1.8 × 10^–4^), respectively (Figure 4A), showing the same tendency when treated by FK866 (Figure 2A). Both 10 mM NAM and 5 μM EX-527 were confirmed to be effective to inhibit SIRT1 deacetylate activity against BMAL1 (Nakahata et al., 2008; Supplementary Figures 2A,B). However, the PARP1 inhibitor, PJ34 (Abdelkarim et al., 2001), did not change both cytoplasmic and nuclear PER2 populations [17.3 ± 3.8% (*p* = 0.56) and 54.6 ± 3.3% (*p* = 0.10), respectively] (Figure 4), and even 10 μM PJ34 was confirmed to be effective to reduce poly-ADP-ribosylation (Supplementary Figure 2C). As FK866 treatment attenuated the amplitude of *Bmal1*-luc circadian oscillation (Figures 1D,E), we further investigated whether SIRT1 deacetylation activity is involved in the regulation of amplitude of *Bmal1*-luc circadian oscillation. NAM or EX-527 treatment attenuated the amplitude of *Bmal1*-luc circadian oscillation (Figures 4B,C,E). Intriguingly, a delayed phase was observed only in cells treated with 10 mM NAM, the concentration at which the enzymatic activity of SIRT1 and also others such as SIRT2 and PARP1 is inhibited (Virág and Szabó, 2002; Peck et al., 2010). However, a delayed phase was not observed in cells treated with 5 μM EX-527, the concentration at which the enzymatic activity of SIRT1 is specifically inhibited (Peck et al., 2010), suggesting that SIRT1 deacetylation activity is involved in the amplitude regulation and other enzymes inhibited by NAM are involved in the phase regulation. *Sirt1*-knockdown cells also demonstrated the attenuated *Bmal1*-luc amplitude, but not a delayed phase (Figure 4D). Knockdown efficiencies against *Sirt1* and circadian oscillation patterns of other *Sirt1* knockdown cell lines are shown in Supplementary Figure 9. These results demonstrate that the NAD^+^/SIRT1 axis regulates PER2 subcellular localization, which further affects the amplitude of the circadian clock.

**FIGURE 5:**
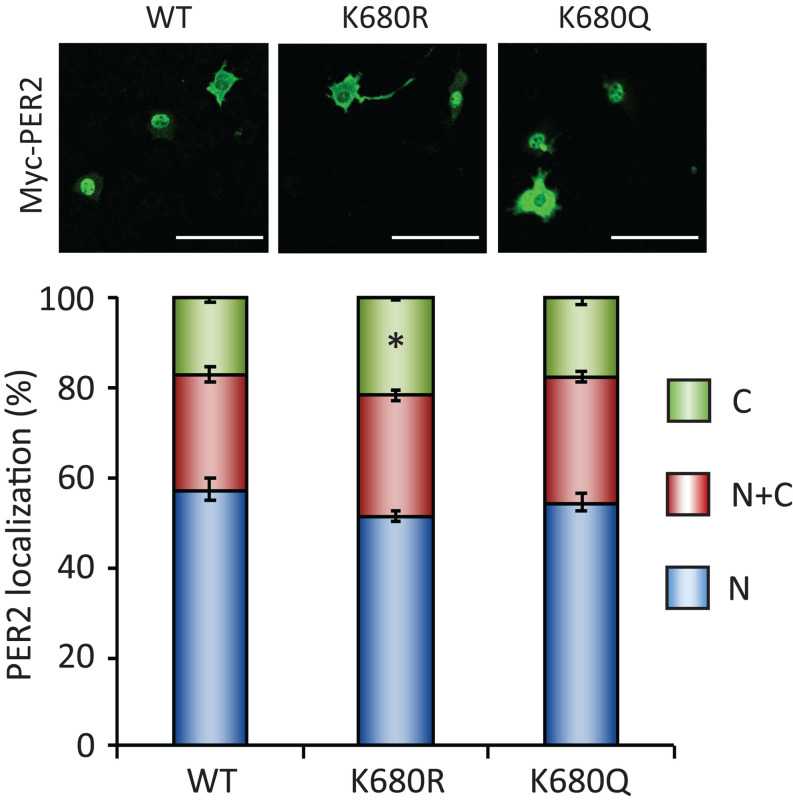
PER2^*K*680^ mutants have no effects on PER2 subcellular localizations. NIH3T3 cells expressing either Myc-PER2-WT (WT), Myc-PER2^*K*680*R*^ (KR) or Myc-PER2^*K*680*Q*^ (KQ) were immunostained with antibody to Myc (top). The representative images were captured by a confocal laser scanning microscope. White scale bars represent 100 μm. (bottom) Subcellular localizations were quantified; N, nucleus; C, cytoplasm; N + C, both nucleus and cytoplasm. The data represented are the mean ± SEM of three independent samples. ^∗^*p* < 0.05, compared to each subcellular localization in WT cells by Student’s two-tailed *t-*test.

To further investigate whether the acetylation status of lysine 680 residue of PER2, where PER2 is acetylated and deacetylated by SIRT1 (Levine et al., 2020), affects its subcellular localization, we analyzed subcellular localizations of lysine 680 residue mutants of PER2 (Figure 5). Non-acetyl mimetic PER2^*K*680*R*^ mutant only slightly increased in the cytoplasmic population of PER2, compared with the cytoplasmic population of PER2^*WT*^ (*p* = 0.010). Moreover, acetyl mimetic PER2^*K*680*Q*^ had no effect on PER2 subcellular localization. These mutant PER2 experiments suggest that K680 of PER2 is not the responsible lysine to regulate subcellular localization by SIRT1, although we cannot deny the possibility that acetyl mimetic mutant of PER2^*K*680*Q*^ does not completely substitute the functions of acetyl-K680 of PER2.

## Discussion

In this study, we found that low intracellular NAD^+^, which was induced by the pharmacological inhibition of NAMPT, promoted PER2 subcellular localization from the nucleus to the cytoplasm and increased and decreased the expressions of E-box- and RORE-regulated circadian genes, respectively, leading to the attenuated *Bmal1*-luc circadian oscillation. Furthermore, we demonstrated that SIRT1 was responsible for NAD^+^-dependent PER2 subcellular regulation, and suggested that lysine 680 residue on PER2 is not responsible for SIRT1-regulated PER2 subcellular localization.

Since several lines of evidence indicate that a decline in NAD^+^ is a hallmark of senescence/aging (Braidy et al., 2011; Yoshino et al., 2011; Canto et al., 2012; Massudi et al., 2012; Camacho-Pereira et al., 2016; Zhang et al., 2016; Khaidizar et al., 2017), in this study, we used NAMPT inhibitors, FK866 and GMX1778, to reduce intracellular NAD^+^, which, we expect, mimics cellular senescence. However, many other physiological events and metabolites such as AMP/ATP ratio and polyamines are known to be changed with senescence/aging (James et al., 2015; Zwighaft et al., 2015). An increase in the AMP/ATP ratio promotes AMP-activated protein kinase (AMPK), which acts as a sensor of the reduced energetic state and further activates catabolic pathways while inhibiting anabolic ones (Hardie, 2003; Garcia and Shaw, 2017). Meanwhile, it has been reported that mTOR, which is an intracellular nutrient sensor for high cellular energy state and associated with autophagy, is also upregulated during senescence (Herranz et al., 2015; Laberge et al., 2015; Nacarelli and Sell, 2017). These signaling molecules have been reported to be involved in the circadian clock. AMPK is a rhythmically expressed kinase and phosphorylates CK1ε, resulting in enhanced phosphorylation and degradation of PER2 (Um et al., 2007; Sahar and Sassone-Corsi, 2012) and CRY1 (Lamia et al., 2011; Sahar and Sassone-Corsi, 2012; Jordan and Lamia, 2013). mTOR perturbation, such as RNAi knockdown or mTOR inhibitors, alters circadian rhythms in fibroblast, SCN, and animal behaviors (Zhang et al., 2009; Ramanathan et al., 2018). Several lines of evidence mentioned here imply that the altered aforementioned signaling pathways during senescence may affect circadian clock properties, although it is largely unknown whether these pathways alter circadian clock properties in senescent cells. We recently demonstrated that altered circadian properties such as period extension and phase delay occur in senescent human cells (Ahmed et al., 2019, 2021). Therefore, we assume that these circadian alterations in senescent cells will be useful indexes to evaluate whether low NAD^+^ or other conditions are sufficient for the circadian clock to mimic senescent condition.

Real-time luciferase monitoring assay revealed that the attenuated amplitude of *Bmal1-*luc oscillations was observed by either NAMPT inhibitors, FK866 and GMX1778; Sirtuins and PARPs broad inhibitor, NAM; SIRT1-specific inhibitor, EX-527; or *Sirt1* knockdown. However, the delayed phase of *Bmal1-*luc oscillations was not observed by SIRT1-specific inhibitor, EX-527, or *Sirt1* knockdown. These results indicate that SIRT1 deacetylation activity is involved in amplitude regulation, but not in phase regulation. This is supported by the report that circadian profiles of *Dbp* and *Per2* in *Sirt1^–/–^* cells showed higher amplitude and no delayed phase (Nakahata et al., 2008). In contrast, delayed phases were observed with FK866, GMX1778, or NAM treatment, suggesting that NAD^+^-dependent enzymes other than SIRT1 are involved in the phase regulation. The investigation to address which enzyme is associated with phase regulation will be needed to understand the mechanisms of how circadian clocks work in senescent cells. Actually, we have recently shown that senescent cells possess altered circadian clocks with a prolonged period and delayed phase (Ahmed et al., 2019, 2021).

In this study, we investigated whether acetylation of lysine 680 residue on PER2 is responsible for PER2 subcellular localization; however, neither acetyl mimetic mutant (K680Q) nor non-acetyl mimetic mutant (K680R) of PER2 changed subcellular localization such as low NAD^+^ or SIRT1 inhibitor-treated conditions. Levine and coworkers recently reported that PER2 is acetylated on K680, which is deacetylated by SRIT1; furthermore, FK866 treatment increases PER2 phosphorylation at numerous sites (Levine et al., 2020). This suggests that acetylation of K680 or other lysine residues of PER2 might be a trigger for subsequent phosphorylation(s), which might determine PER2 subcellular localization. Our current results cannot deny the possibility that acetyl mimetic mutant PER2^*K*680*Q*^ may not trigger subsequent phosphorylation(s) because of differences of three-dimensional structures triggered by the acetylated form of lysine residue and substituted glutamine residue. Further experiments including structural biological experiments will be needed to elucidate which lysine residue is responsible for subcellular localization of PER2 regulated by SIRT1.

Our current results suggest that PER/CRY-dependent repression against E-box-regulated genes might be dependent on the NAD^+^/SIRT1 axis. Intriguingly, we have reported that SIRT1 deacetylates lysine 9/14 residues of histone H3 on the promoters of E-box-regulated genes to repress their expressions epigenetically (Nakahata et al., 2008). These results imply that the NAD^+^/SIRT1 axis coordinates the transcriptional repression of E-box-regulated genes by different ways. In addition to the coordinated mechanisms for E-box-regulated genes, RORE-regulated genes might also be coordinated by the NAD^+^/SIRT1 axis. It has been reported that NAD^+^ activates *Bmal1* transcription by SIRT1 through the deacetylation of PGC1α (Chang and Guarente, 2014), while we demonstrated in this study that the low NAD^+^ condition increased *Reb-erb*α/β which in turn might repress *Bmal1* gene expression. These coordinated *Bmal1* gene regulations by the NAD^+^/SIRT1 axis presumably regulate the amplitude of *Bmal1* circadian oscillation, namely the low NAD^+^ condition or decreased SIRT1 activity attenuates the amplitude of *Bmal1* circadian oscillation. Therefore, these coordinated transcriptional regulations of E-box- and RORE-regulated circadian genes by the NAD^+^/SIRT1 axis might interlock circadian negative feedback loops.

In addition to our finding that low NAD^+^ retains PER2 in the cytoplasm, some studies have demonstrated that the NAD^+^/SIRT1 axis regulates PER2 properties; PER2 in *Sirt1^–/–^* cells is more stable (Asher et al., 2008) and localizes predominantly in the nucleus (Levine et al., 2020), while PER2 in nicotinamide-treated cells localizes predominantly in the cytoplasm (Miki et al., 2012). Our pharmacologically conducted results were consistent with the results pharmacologically performed by Miki and colleagues, but not with the results performed genetically. These conflicting results by pharmacological (acute) and genetical (chronic) approaches suggest the possibility that responses of molecular clock are different at early and chronic senescence/aging phases. This possibility is worth verifying so further experiments using different stages of senescent cells/aging animals will be needed to reveal this possibility.

## Data Availability Statement

The raw data supporting the conclusions of this article will be made available by the authors, without undue reservation.

## Author Contributions

AA performed and analyzed the experiments. YN designed the research and wrote the manuscript. TS, YuF, HY, and YoF provided the reagents. KS assisted with the experiments and reviewed the manuscript. TM and YB designed the research and reviewed the manuscript. All authors contributed to the article and approved the submitted version.

## Conflict of Interest

TS and YF were employed by the Mitsubishi Corporation Life Sciences Limited. The remaining authors declare that the research was conducted in the absence of any commercial or financial relationships that could be construed as a potential conflict of interest.

## Supplementary Material

Supplementary Figure 1 Representative Immunofluorescence images. N, nucleus; C, cytoplasm; N + C, nuclear and cytoplasm. We interpreted a result for nuclear localization of PER2/CRY1 (N) as positive when fluorescence intensity (Alexa Fluor Plus 488) of PER2/CRY1 signal that overlapped with Hoechst 33342 signal was higher than the fluorescence intensity of PER2/CRY1 in the cytoplasm Meanwhile, we interpreted a result for cytoplasmic localization of PER2/CRY1 (C) as positive when PER2/CRY1 signal that did not overlap with Hoechst 33342 signal exhibited higher fluorescence intensity than PER2/CRY1 signal intensity that overlapped with Hoechst 33342 signal. Finally, we interpreted a result for nuclear and cytoplasm localization of PER2/CRY1 (N + C) as positive when PER2/CRY1 fluorescence signal at both nucleus and cytoplasm had the same intensities.

Supplementary Figure 2 Confirmations of inhibitors by western blotting. **(A)** NIH3T3 cells were treated with FK866 or EX-527 at indicated concentrations for 24 h. Total cell extracts were immunoblotted with antibodies against acetylated form of BMAL1 (Ac-BMAL1) or BMAL1. **(B)** NIH3T3 cells were treated with 10 mM NAM for 24 h. Total cell extracts from NIH3T3 (left and middle lanes) and mouse embryonic fibroblast derived from *Sirt1*^–/–^ without any treatment (right lane) were immunoblotted with antibodies against acetylated form of BMAL1 (Ac-BMAL1) or BMAL1. **(C)** NIH3T3 cells were treated with 10 μM PJ34 for 24 h. Total cell extracts were immunoblotted with antibodies against poly ADP-ribosylation (PAR/pADPr), PARP1 or TUBLIN.

Supplementary Figure 3 GMX1778 decreases in NAD^+^ in cells. **(A)** NAD^+^ amount after 24 h treatment of GMX1778 at indicated concentrations were measured by HPLC. NAD^+^ amount was normalized by protein amount. **(B)** NAD^+^ amount after 24 h treatment of 75 nM GMX1778 with or without 1mM NMN were measured by HPLC. NAD^+^ amount was normalized by protein amount. **(C)** *Bmal1*-luc oscillation patterns treated with GMX1778 with or without NMN were monitored by using a real-time luciferase monitoring system. **(D)** Relative amplitudes were analyzed. The value of control (ctrl) was set to 1. All data represented in Supplementary Figure 3 are the mean ± SEM of independent three samples. ^∗∗^*p* < 0.01 and ^∗∗∗^*p* < 0.001, compared to each of the untreated control by Student’s two-tailed *t*-test, n.s., means not significant.

Supplementary Figure 4 Effects of FK866 or GMX1778 on intracellular NAD^+^ amount. NIH3T3 were treated with NAMPT inhibitors, FK866 or GMX1778, throughout this experiment.

Supplementary Figure 5 FK866 has no effect on stabilities of clock proteins. NIH3T3 cells expressing Myc-CLOCK **(A)**, Myc-BMAL1 **(B)**, Myc-PER2 **(C),** or Myc-CRY1 **(D)** were treated with cycloheximide (CHX) at 100 μg/ml. At indicated times, cells were lysed and protein extracts were immunoblotted with antibody to Myc (top) and antibody to Tubulin (middle) as loading control. Cells were either untreated (ctrl) or treated with 7.5 nM FK866. The results are representative of three independent experiments. (Bottom) The immunoblots were quantified by densitometric analysis. The graph shows the percentage of protein amount relative to time 0 (100%). Myc-tagged proteins were normalized by each Tubulin amount.

Supplementary Figure 6 FK866 has no effect on subcellular localizations of CLOCK, BMAL1, RORα/γ and REV-ERVα/β. NIH3T3 cells expressing Myc-CLOCK **(A)**, Myc-BMAL1 **(B)**, Flag-RORα **(C)**, Flag-RORγ **(D)**, Flag-REV-ERBα **(E)**, or Flag-REV-ERBβ **(F)** were immunostained with antibody to Myc or DDDDK (top). Cells were untreated (ctrl), treated with 7.5 nM FK866 (FK) or 7.5 nM FK866 & 1 mM NMN (FK + NMN). The representative images were captured by a confocal laser scanning microscope. (bottom) Subcellular localizations were quantified; N, nucleus; C, cytoplasm; N + C, both nucleus and cytoplasm. White scale bars represent 100 μm. The data represented are the mean ± SEM of three independent samples.

Supplementary Figure 7 GMX1778 changes PER2 subcellular localization, which is abrogated by co-treatment with NMN. (top) NIH3T3 cells expressing indicated Myc-PER2 were immunostained with antibody to Myc. Cells were untreated (ctrl), treated with 75 nM GMX1778 (GMX) or 75 nM GMX1778 & 1 mM NMN (GMX + NMN). The representative images were captured by a confocal laser scanning microscope. White scale bars represent 100 μm. (bottom) Subcellular localizations were quantified; N, nucleus; C, cytoplasm; N + C, both nucleus and cytoplasm. The data represented are the mean ± SEM of three independent samples. ^∗∗∗^*p* < 0.001, compared to each subcellular localization in control cells by Student’s two-tailed *t-*test.

Supplementary Figure 8 CRY1 did not alter PER2 subcellular localization. **(A)** Subcellular localizations of either PER2-WT, PER2-NLS, or PER2-NES were quantified; N, nucleus; C, cytoplasm; N + C, both nucleus and cytoplasm. **(B)** Subcellular localizations of either PER2-WT, PER2-NLS, or PER2-NES were quantified when CRY1 was co-expressed. The data represented are the mean ± SEM of three independent samples. ^∗^*p* < 0.05, ^∗∗^*p* < 0.01, and ^∗∗∗^*p* < 0.001, compared to each subcellular localization in cells expressing PER2-WT by Student’s two-tailed *t-*test.

Supplementary Figure 9 *Sirt1* knockdown attenuates the amplitude of *Bmal1*-luc oscillation. **(A)** Knockdown efficiencies against *Sirt1* were analyzed by qPCR. *Sirt1* amount in control (scramble) was set to 1. The data represented are the mean ± SEM of independent three samples. **(B)** *Bmal1*-luc oscillation patterns in *Sirt1* knockdown (sh*Sirt1* #1 or #2) NIH3T3 cells were monitored by using a real-time luciferase monitoring system. **(C)** Relative amplitudes were analyzed. The value of control (ctrl) was set to 1. The data represented are the mean ± SEM of independent 8, 3, or 3 samples for control, sh*Sirt1* #1 or #2, respectively. ^∗∗^*p* < 0.01, compared to control cells by Student’s two-tailed *t*-test, n.s., means not significant.

Supplementary Table 1 Primer sequences for qPCR.

Supplementary Table 2 Oligonucleotide sequences for knockdown against *Sirt1*.
