# Supplementary material for: Attenuated SIRT1 Activity Leads to PER2 Cytoplasmic Localization and Dampens the Amplitude of Bmal1 Promoter-Driven Circadian Oscillation
Source: Front Neurosci. 2021 May 24;15:647589. doi: 10.3389/fnins.2021.647589 (PMC8180908; doi:10.3389/fnins.2021.647589)
Supplement: Supplementary Figure 1 — Representative Immunofluorescence images. N, nucleus; C, cytoplasm; N + C, nuclear and cytoplasm. We interpreted a result for nuclear localization of PER2/CRY1 (N) as positive when fluorescence intensity (Alexa Fluor Plus 488) of PER2/CRY1 signal that overlapped with Hoechst 33342 signal was higher than the fluorescence intensity of PER2/CRY1 in the cytoplasm Meanwhile, we interpreted a result for cytoplasmic localization of PER2/CRY1 (C) as positive when PER2/CRY1 signal that did not overlap with Hoechst 33342 signal exhibited higher fluorescence intensity than PER2/CRY1 signal intensity that overlapped with Hoechst 33342 signal. Finally, we interpreted a result for nuclear and cytoplasm localization of PER2/CRY1 (N + C) as positive when PER2/CRY1 fluorescence signal at both nucleus and cytoplasm had the same intensities. [file Presentation_1.pptx]

## Slide 1
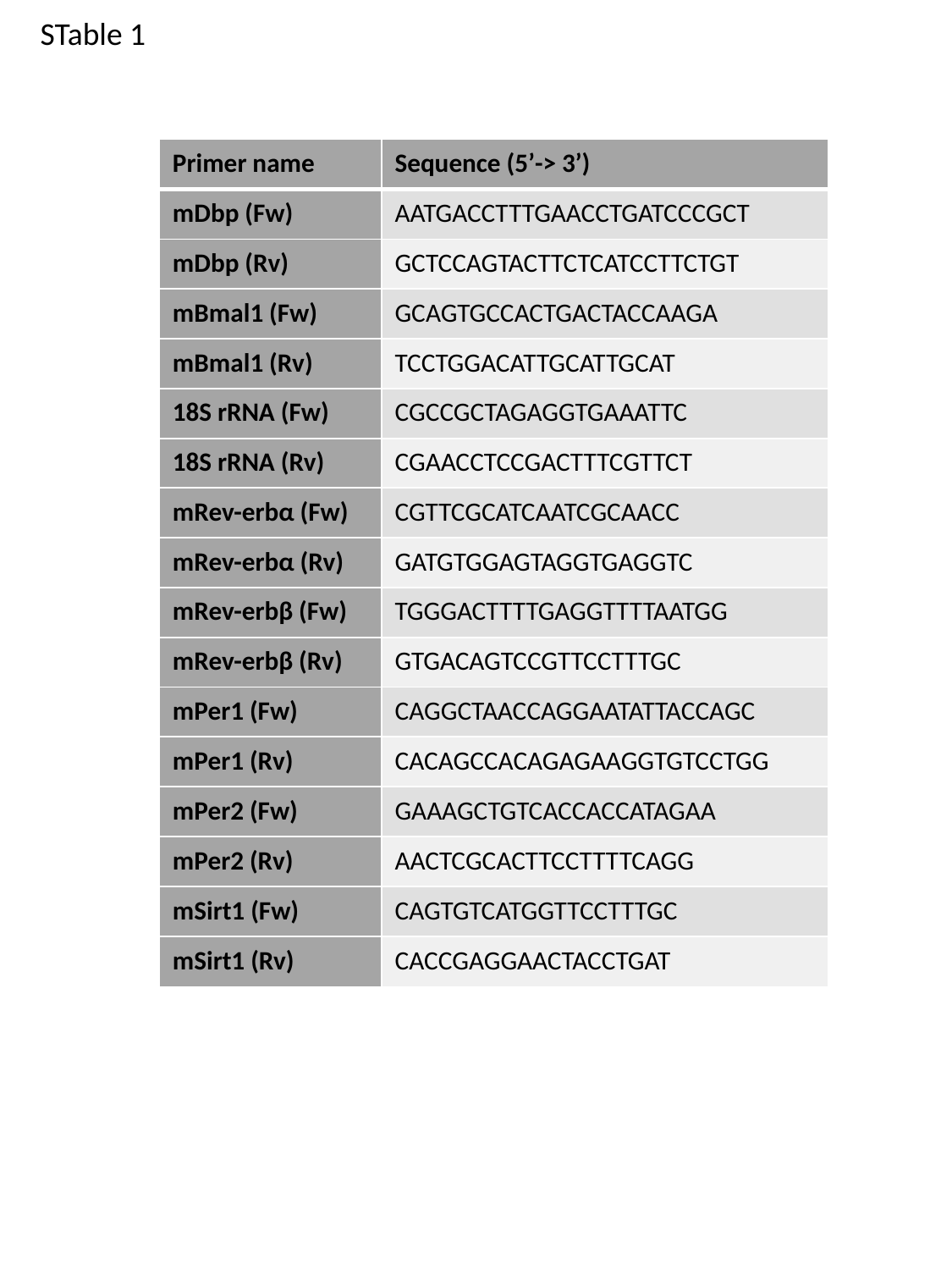

STable 1
| Primer name | Sequence (5’-> 3’) |
| --- | --- |
| mDbp (Fw) | AATGACCTTTGAACCTGATCCCGCT |
| mDbp (Rv) | GCTCCAGTACTTCTCATCCTTCTGT |
| mBmal1 (Fw) | GCAGTGCCACTGACTACCAAGA |
| mBmal1 (Rv) | TCCTGGACATTGCATTGCAT |
| 18S rRNA (Fw) | CGCCGCTAGAGGTGAAATTC |
| 18S rRNA (Rv) | CGAACCTCCGACTTTCGTTCT |
| mRev-erbα (Fw) | CGTTCGCATCAATCGCAACC |
| mRev-erbα (Rv) | GATGTGGAGTAGGTGAGGTC |
| mRev-erbβ (Fw) | TGGGACTTTTGAGGTTTTAATGG |
| mRev-erbβ (Rv) | GTGACAGTCCGTTCCTTTGC |
| mPer1 (Fw) | CAGGCTAACCAGGAATATTACCAGC |
| mPer1 (Rv) | CACAGCCACAGAGAAGGTGTCCTGG |
| mPer2 (Fw) | GAAAGCTGTCACCACCATAGAA |
| mPer2 (Rv) | AACTCGCACTTCCTTTTCAGG |
| mSirt1 (Fw) | CAGTGTCATGGTTCCTTTGC |
| mSirt1 (Rv) | CACCGAGGAACTACCTGAT |

## Slide 2
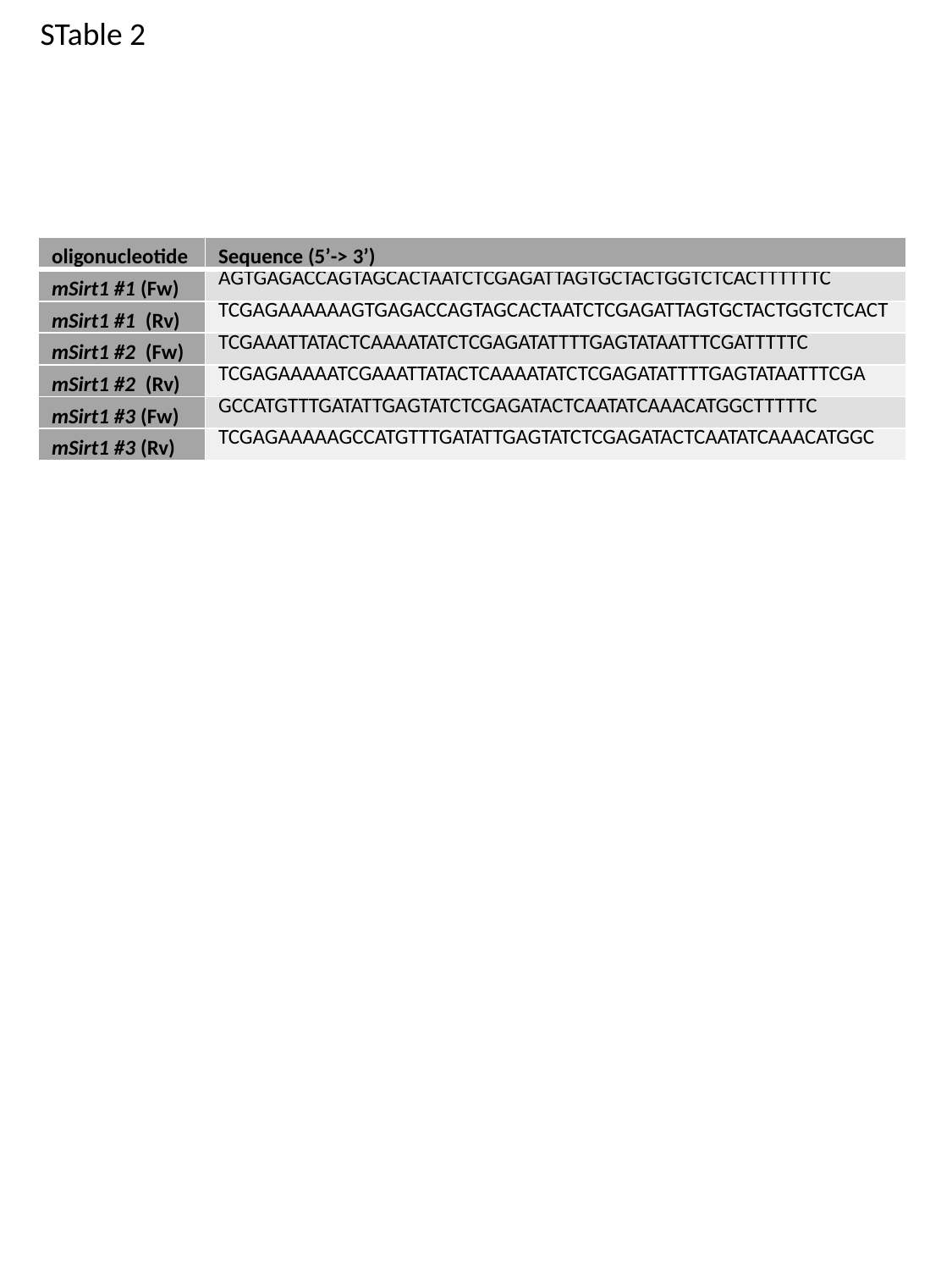

STable 2
| oligonucleotide | Sequence (5’-> 3’) |
| --- | --- |
| mSirt1 #1 (Fw) | AGTGAGACCAGTAGCACTAATCTCGAGATTAGTGCTACTGGTCTCACTTTTTTC |
| mSirt1 #1 (Rv) | TCGAGAAAAAAGTGAGACCAGTAGCACTAATCTCGAGATTAGTGCTACTGGTCTCACT |
| mSirt1 #2 (Fw) | TCGAAATTATACTCAAAATATCTCGAGATATTTTGAGTATAATTTCGATTTTTC |
| mSirt1 #2 (Rv) | TCGAGAAAAATCGAAATTATACTCAAAATATCTCGAGATATTTTGAGTATAATTTCGA |
| mSirt1 #3 (Fw) | GCCATGTTTGATATTGAGTATCTCGAGATACTCAATATCAAACATGGCTTTTTC |
| mSirt1 #3 (Rv) | TCGAGAAAAAGCCATGTTTGATATTGAGTATCTCGAGATACTCAATATCAAACATGGC |

## Slide 3
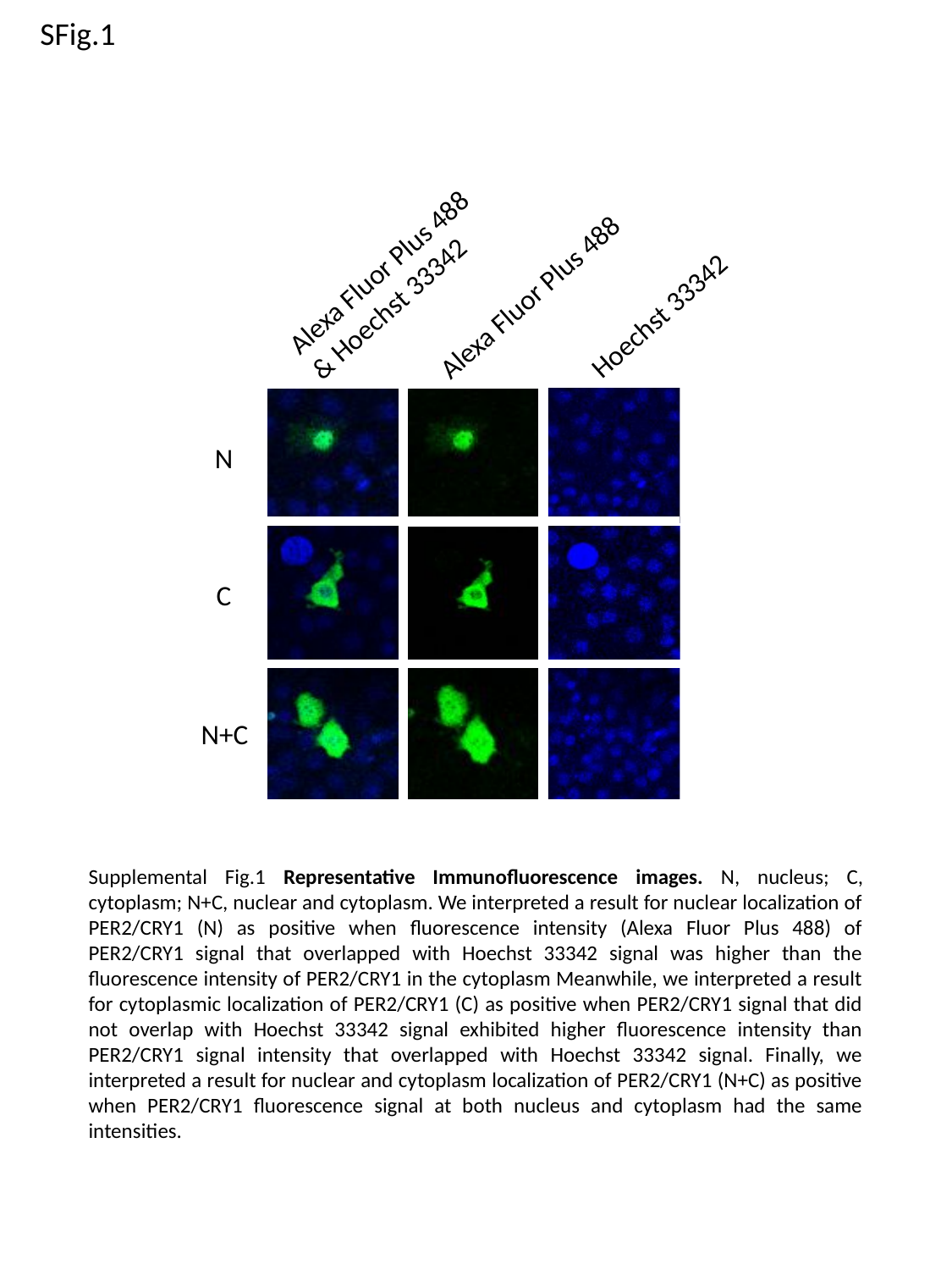

SFig.1
Alexa Fluor Plus 488
& Hoechst 33342
Alexa Fluor Plus 488
Hoechst 33342
N
C
N+C
Supplemental Fig.1 Representative Immunofluorescence images. N, nucleus; C, cytoplasm; N+C, nuclear and cytoplasm. We interpreted a result for nuclear localization of PER2/CRY1 (N) as positive when fluorescence intensity (Alexa Fluor Plus 488) of PER2/CRY1 signal that overlapped with Hoechst 33342 signal was higher than the fluorescence intensity of PER2/CRY1 in the cytoplasm Meanwhile, we interpreted a result for cytoplasmic localization of PER2/CRY1 (C) as positive when PER2/CRY1 signal that did not overlap with Hoechst 33342 signal exhibited higher fluorescence intensity than PER2/CRY1 signal intensity that overlapped with Hoechst 33342 signal. Finally, we interpreted a result for nuclear and cytoplasm localization of PER2/CRY1 (N+C) as positive when PER2/CRY1 fluorescence signal at both nucleus and cytoplasm had the same intensities.

## Slide 4
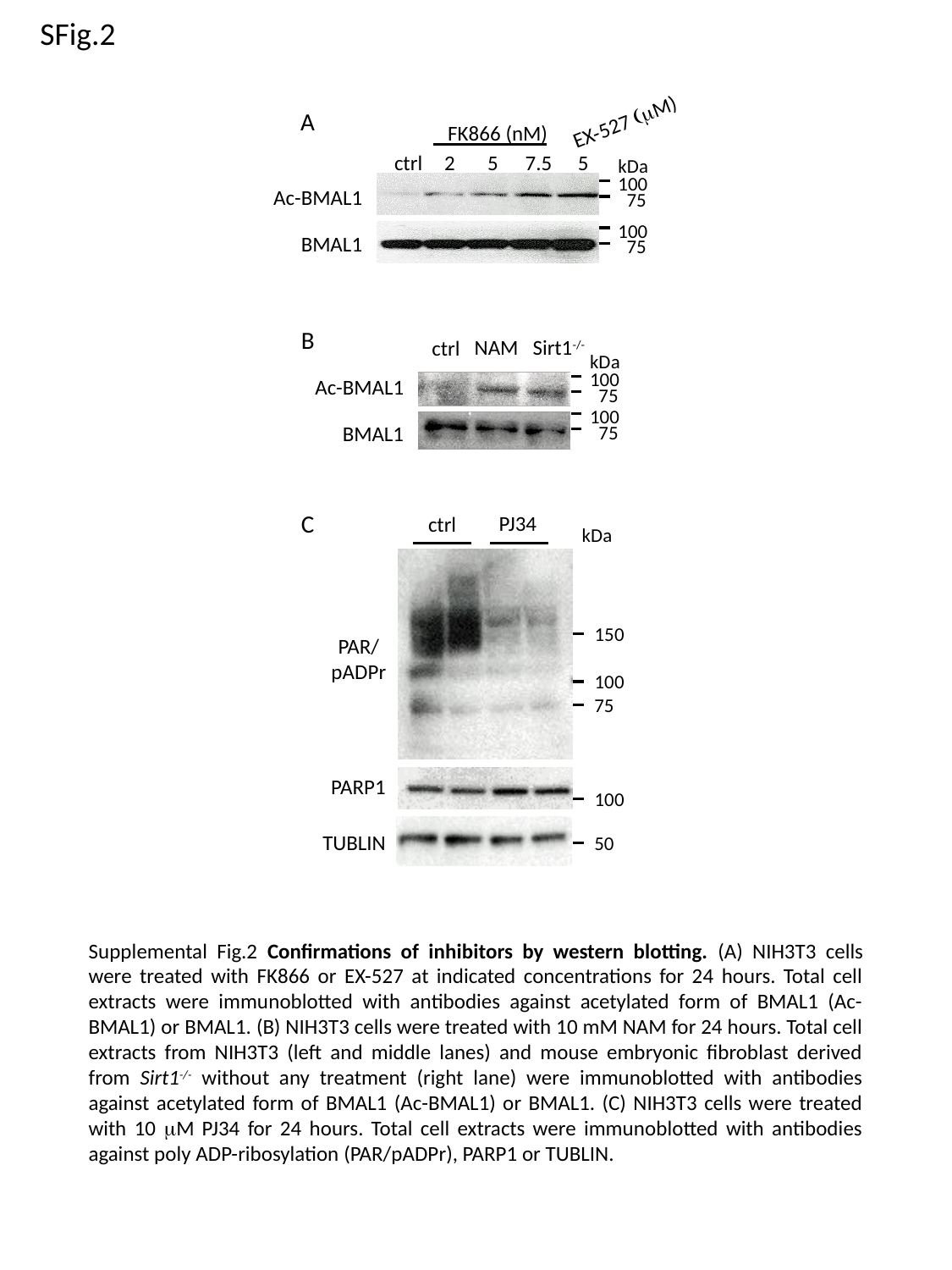

SFig.2
A
EX-527 (mM)
FK866 (nM)
ctrl
2
5
7.5
5
kDa
100
Ac-BMAL1
75
100
BMAL1
75
B
Sirt1-/-
NAM
ctrl
kDa
100
Ac-BMAL1
75
100
75
BMAL1
C
PJ34
ctrl
kDa
150
PAR/
pADPr
100
75
PARP1
100
TUBLIN
50
Supplemental Fig.2 Confirmations of inhibitors by western blotting. (A) NIH3T3 cells were treated with FK866 or EX-527 at indicated concentrations for 24 hours. Total cell extracts were immunoblotted with antibodies against acetylated form of BMAL1 (Ac-BMAL1) or BMAL1. (B) NIH3T3 cells were treated with 10 mM NAM for 24 hours. Total cell extracts from NIH3T3 (left and middle lanes) and mouse embryonic fibroblast derived from Sirt1-/- without any treatment (right lane) were immunoblotted with antibodies against acetylated form of BMAL1 (Ac-BMAL1) or BMAL1. (C) NIH3T3 cells were treated with 10 mM PJ34 for 24 hours. Total cell extracts were immunoblotted with antibodies against poly ADP-ribosylation (PAR/pADPr), PARP1 or TUBLIN.

## Slide 5
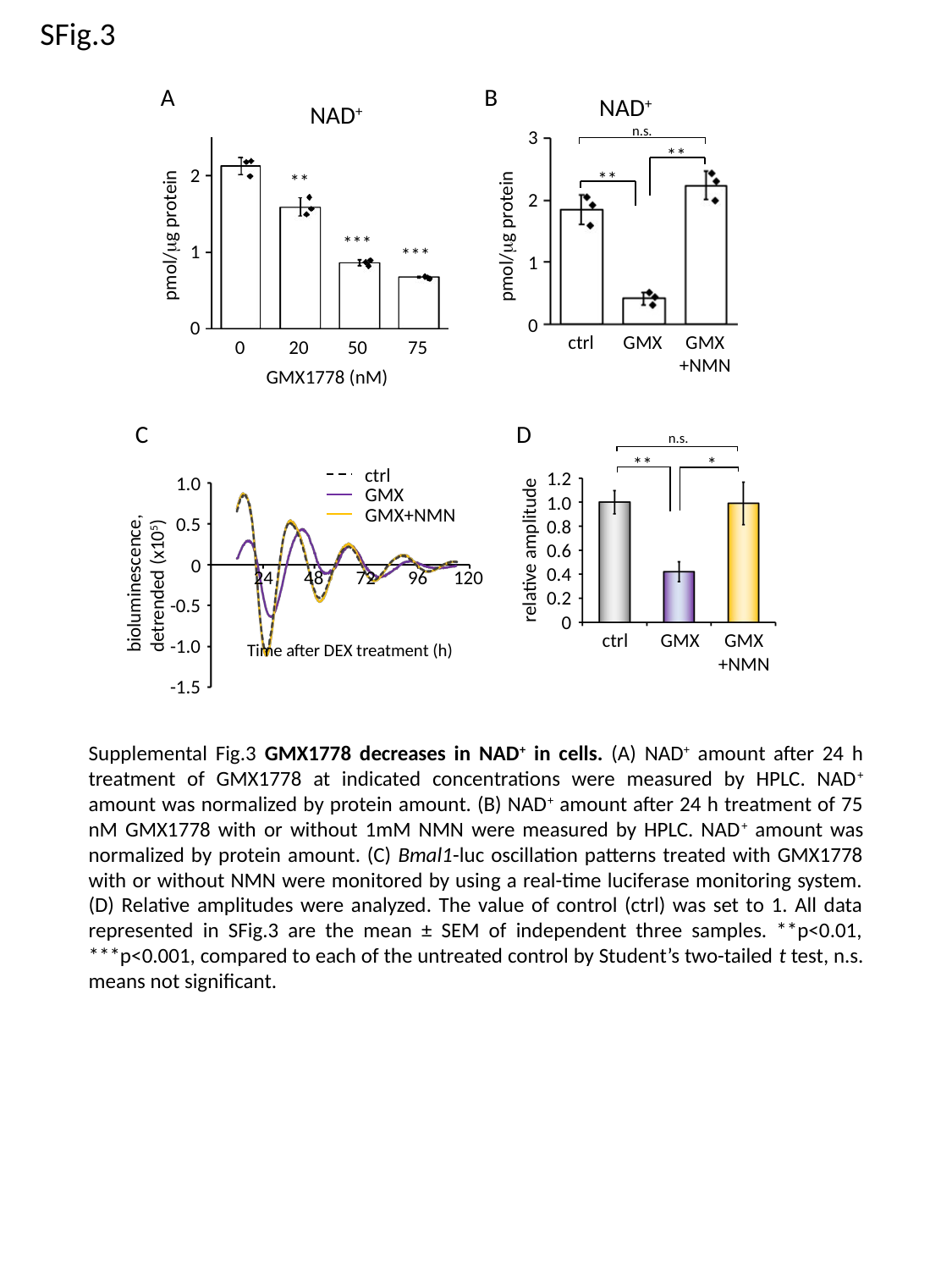

SFig.3
A
B
NAD+
3
2
pmol/mg protein
1
0
ctrl
GMX
GMX
+NMN
n.s.
**
**
NAD+
2
pmol/mg protein
1
0
0
20
50
75
GMX1778 (nM)
**
***
***
C
D
n.s.
**
*
1.2
1.0
0.8
0.6
relative amplitude
0.4
0.2
0
ctrl
GMX
GMX
+NMN
ctrl
GMX
GMX+NMN
1.0
0.5
0
24
48
72
96
120
-0.5
-1.0
Time after DEX treatment (h)
-1.5
bioluminescence,
detrended (x105)
Supplemental Fig.3 GMX1778 decreases in NAD+ in cells. (A) NAD+ amount after 24 h treatment of GMX1778 at indicated concentrations were measured by HPLC. NAD+ amount was normalized by protein amount. (B) NAD+ amount after 24 h treatment of 75 nM GMX1778 with or without 1mM NMN were measured by HPLC. NAD+ amount was normalized by protein amount. (C) Bmal1-luc oscillation patterns treated with GMX1778 with or without NMN were monitored by using a real-time luciferase monitoring system. (D) Relative amplitudes were analyzed. The value of control (ctrl) was set to 1. All data represented in SFig.3 are the mean ± SEM of independent three samples. **p<0.01, ***p<0.001, compared to each of the untreated control by Student’s two-tailed t test, n.s. means not significant.

## Slide 6
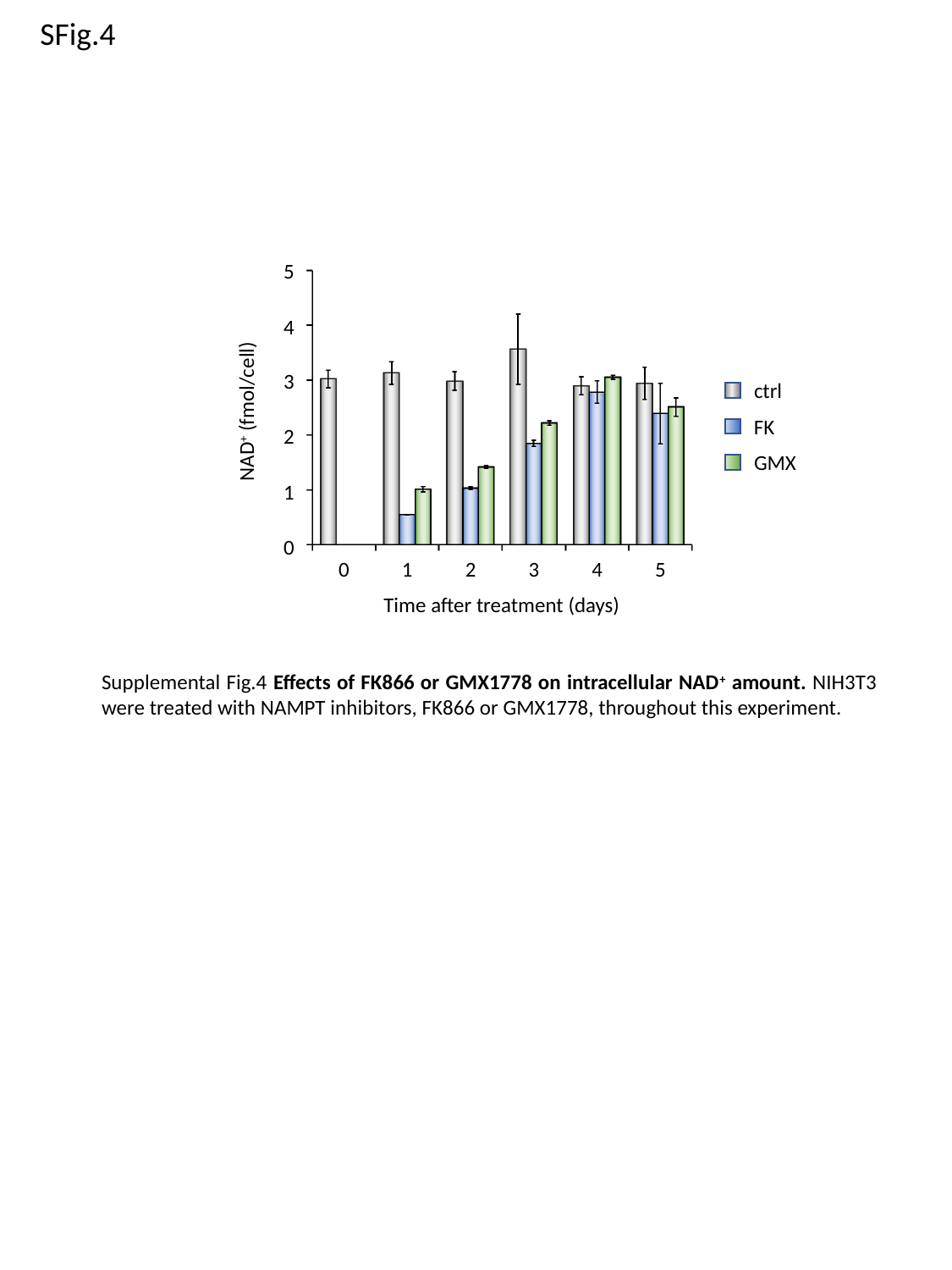

SFig.4
ctrl
FK
GMX
5
4
3
NAD+ (fmol/cell)
2
1
0
0
1
2
3
4
5
Time after treatment (days)
Supplemental Fig.4 Effects of FK866 or GMX1778 on intracellular NAD+ amount. NIH3T3 were treated with NAMPT inhibitors, FK866 or GMX1778, throughout this experiment.

## Slide 7
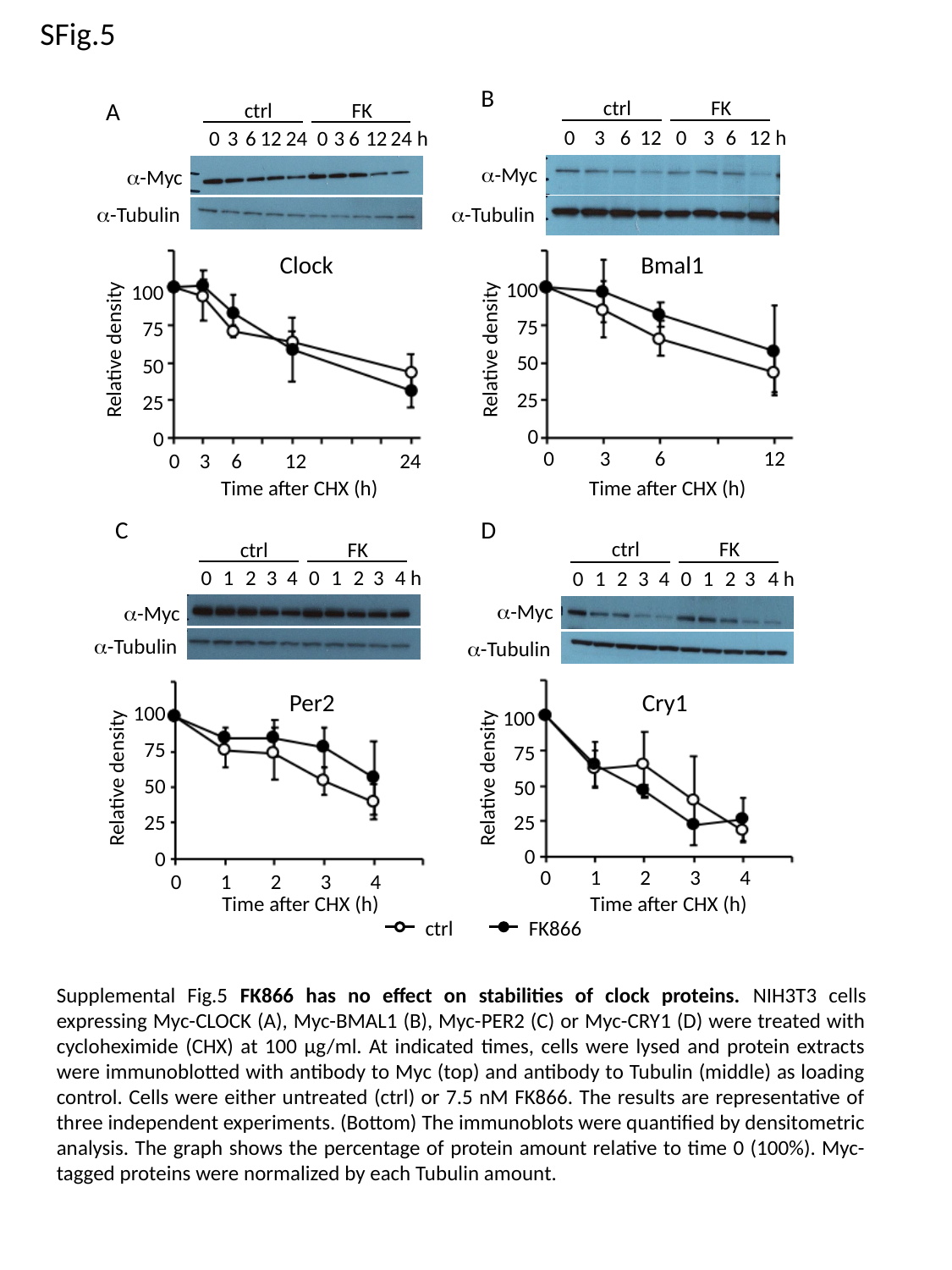

SFig.5
B
ctrl
FK
0
3
6
12
0
3
6
12 h
a-Myc
a-Tubulin
100
75
50
25
0
0
3
6
12
A
ctrl
FK
0
3
6
12
24
0
3
6
12
24 h
a-Myc
a-Tubulin
100
75
50
25
0
0
3
6
12
24
Clock
Bmal1
Relative density
Relative density
Time after CHX (h)
Time after CHX (h)
C
D
ctrl
FK
0
1
2
3
4
0
1
2
3
4 h
a-Myc
a-Tubulin
100
75
50
25
0
0
1
2
3
4
ctrl
FK
0
1
2
3
4
0
1
2
3
4 h
a-Myc
a-Tubulin
100
75
50
25
0
0
1
2
3
4
Per2
Cry1
Relative density
Relative density
Time after CHX (h)
Time after CHX (h)
ctrl
FK866
Supplemental Fig.5 FK866 has no effect on stabilities of clock proteins. NIH3T3 cells expressing Myc-CLOCK (A), Myc-BMAL1 (B), Myc-PER2 (C) or Myc-CRY1 (D) were treated with cycloheximide (CHX) at 100 μg/ml. At indicated times, cells were lysed and protein extracts were immunoblotted with antibody to Myc (top) and antibody to Tubulin (middle) as loading control. Cells were either untreated (ctrl) or 7.5 nM FK866. The results are representative of three independent experiments. (Bottom) The immunoblots were quantified by densitometric analysis. The graph shows the percentage of protein amount relative to time 0 (100%). Myc-tagged proteins were normalized by each Tubulin amount.

## Slide 8
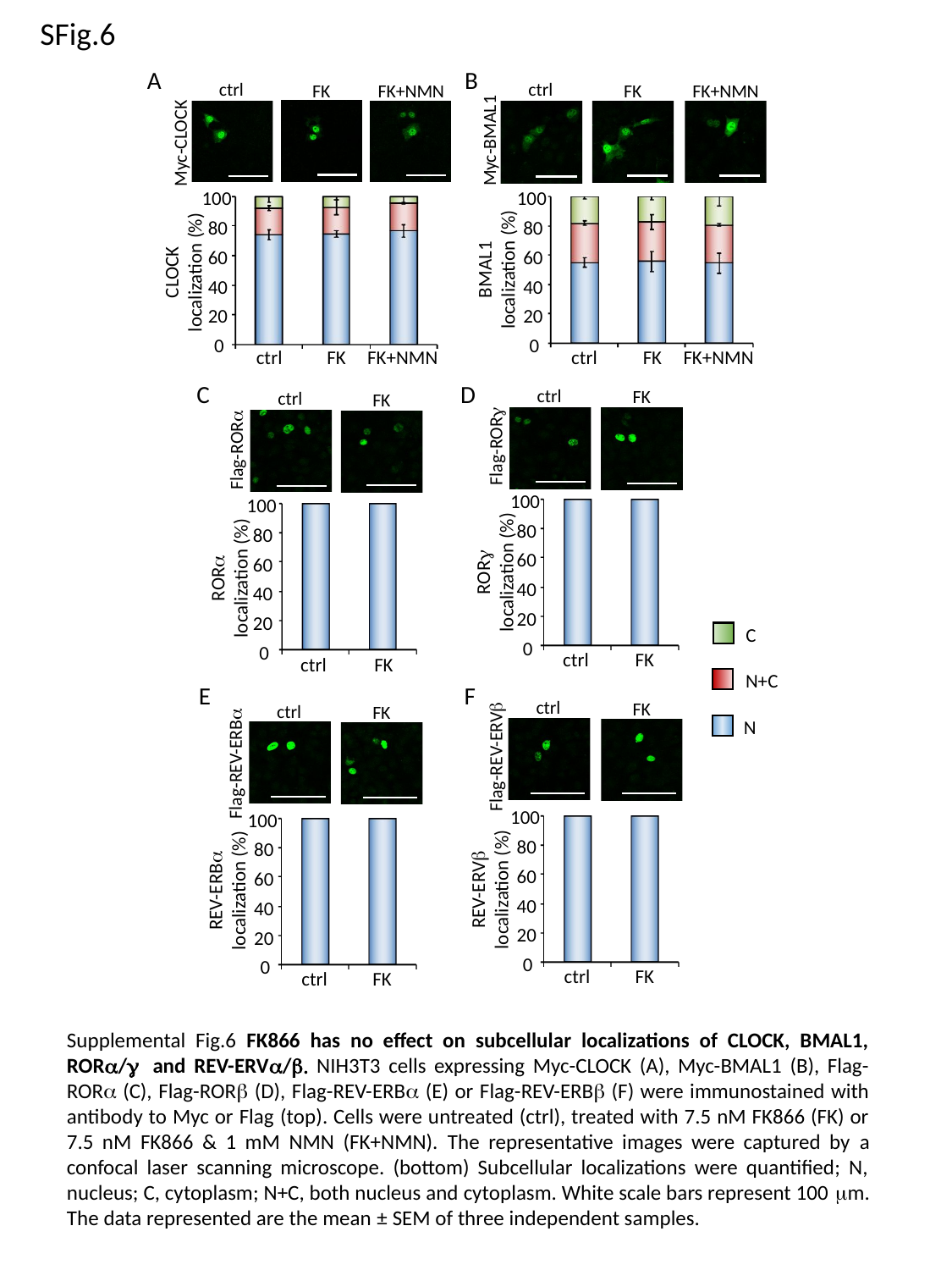

SFig.6
A
B
ctrl
FK+NMN
FK
Myc-BMAL1
100
80
60
BMAL1
localization (%)
40
20
0
ctrl
FK
FK+NMN
ctrl
FK+NMN
FK
Myc-CLOCK
100
80
60
CLOCK
localization (%)
40
20
0
FK
FK+NMN
ctrl
C
D
ctrl
FK
ctrl
FK
Flag-RORg
100
80
60
RORg
localization (%)
40
20
0
ctrl
FK
Flag-RORa
100
80
60
RORa
localization (%)
40
20
0
ctrl
FK
C
N+C
N
E
F
Flag-REV-ERVb
100
80
60
REV-ERVb
localization (%)
40
20
0
FK
ctrl
ctrl
FK
ctrl
Flag-REV-ERBa
100
80
60
REV-ERBa
localization (%)
40
20
0
ctrl
FK
FK
Supplemental Fig.6 FK866 has no effect on subcellular localizations of CLOCK, BMAL1, RORa/g and REV-ERVa/b. NIH3T3 cells expressing Myc-CLOCK (A), Myc-BMAL1 (B), Flag-RORa (C), Flag-RORb (D), Flag-REV-ERBa (E) or Flag-REV-ERBb (F) were immunostained with antibody to Myc or Flag (top). Cells were untreated (ctrl), treated with 7.5 nM FK866 (FK) or 7.5 nM FK866 & 1 mM NMN (FK+NMN). The representative images were captured by a confocal laser scanning microscope. (bottom) Subcellular localizations were quantified; N, nucleus; C, cytoplasm; N+C, both nucleus and cytoplasm. White scale bars represent 100 mm. The data represented are the mean ± SEM of three independent samples.

## Slide 9
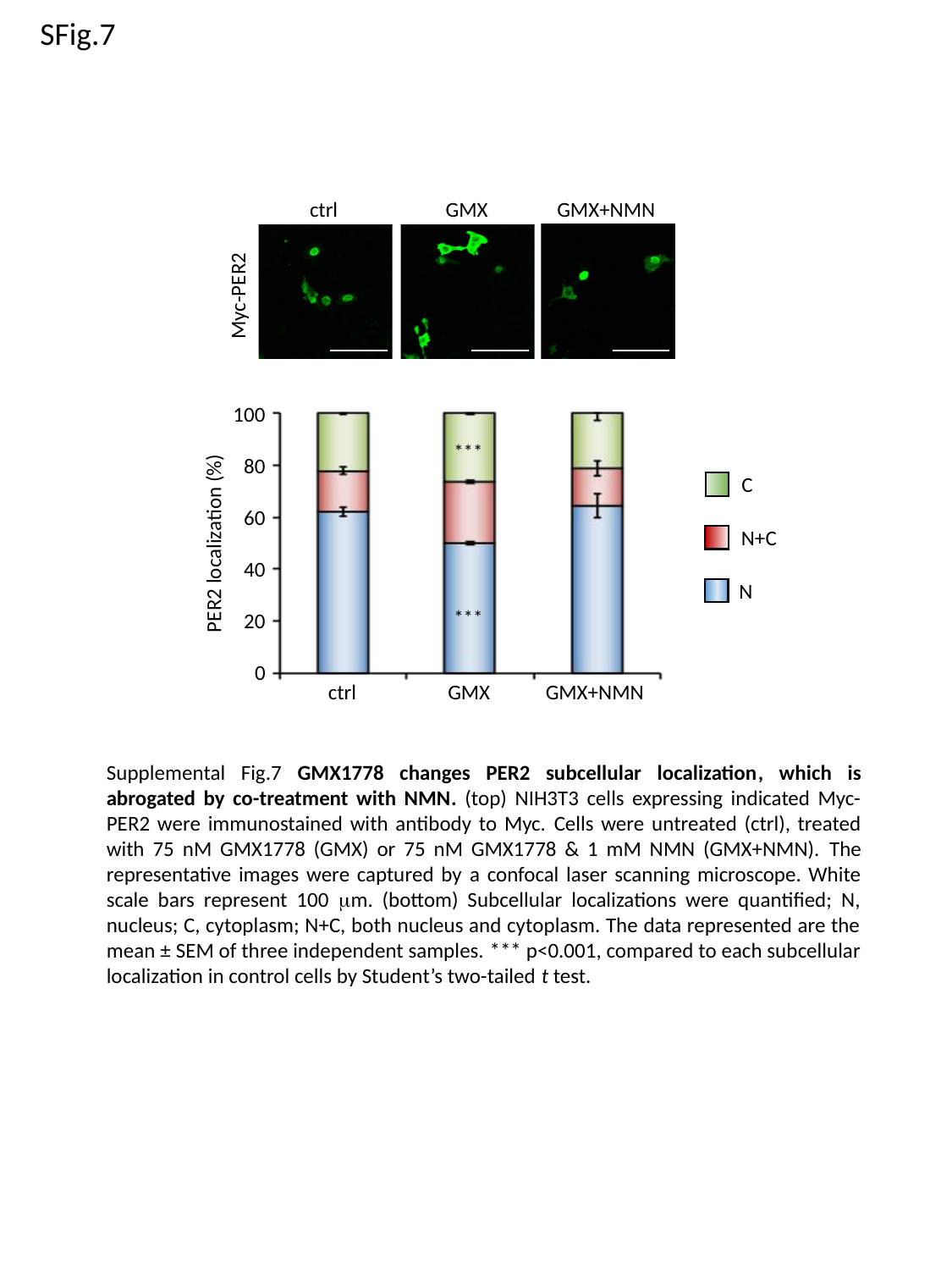

SFig.7
ctrl
GMX
GMX+NMN
Myc-PER2
100
80
C
N+C
N
60
PER2 localization (%)
40
20
0
ctrl
GMX
GMX+NMN
***
***
Supplemental Fig.7 GMX1778 changes PER2 subcellular localization, which is abrogated by co-treatment with NMN. (top) NIH3T3 cells expressing indicated Myc-PER2 were immunostained with antibody to Myc. Cells were untreated (ctrl), treated with 75 nM GMX1778 (GMX) or 75 nM GMX1778 & 1 mM NMN (GMX+NMN). The representative images were captured by a confocal laser scanning microscope. White scale bars represent 100 mm. (bottom) Subcellular localizations were quantified; N, nucleus; C, cytoplasm; N+C, both nucleus and cytoplasm. The data represented are the mean ± SEM of three independent samples. *** p<0.001, compared to each subcellular localization in control cells by Student’s two-tailed t test.

## Slide 10
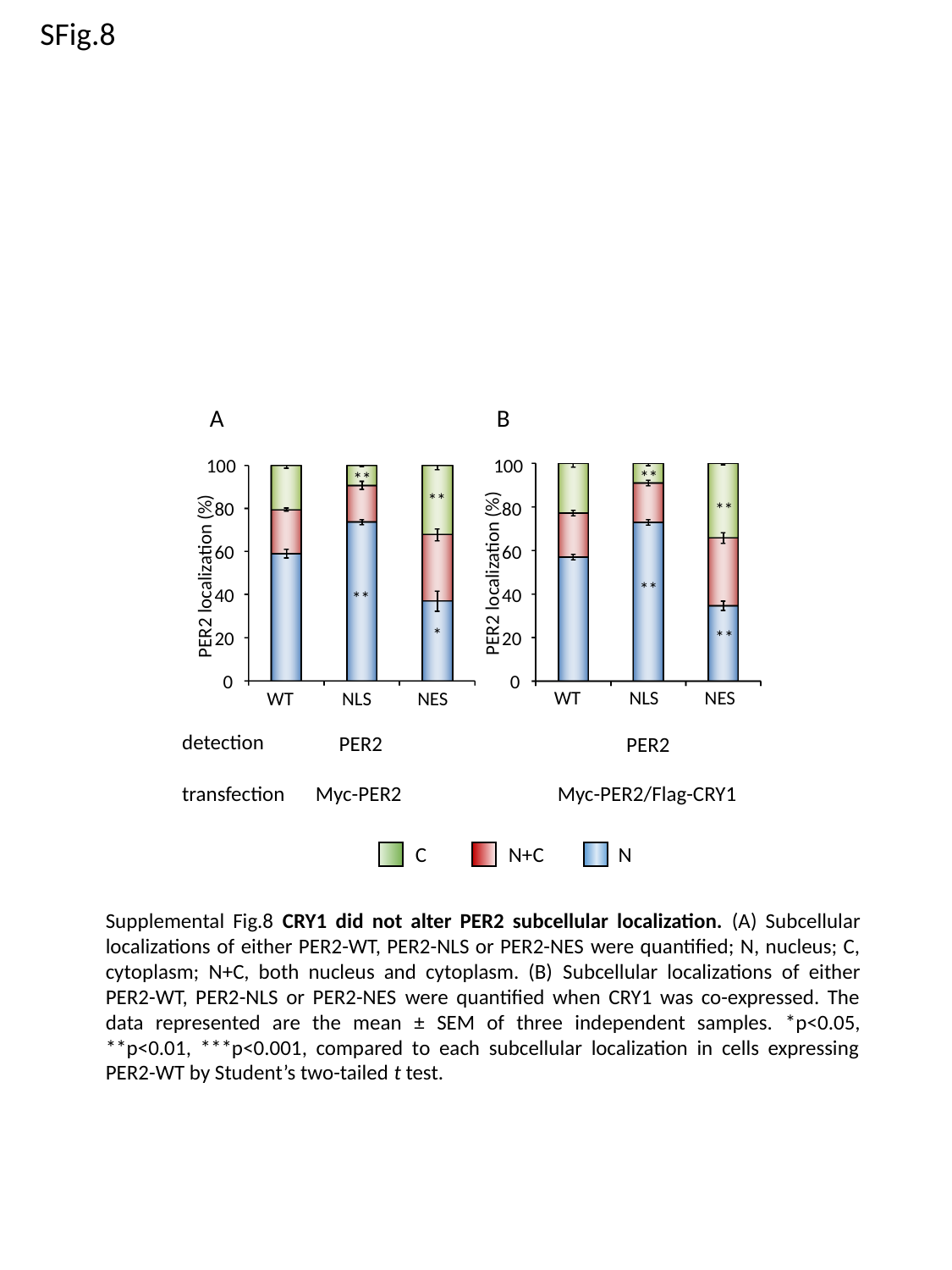

SFig.8
A
B
100
80
60
PER2 localization (%)
40
20
0
WT
NLS
NES
100
80
60
PER2 localization (%)
40
20
0
WT
NLS
NES
**
**
**
**
**
**
*
**
detection
PER2
PER2
transfection
Myc-PER2
Myc-PER2/Flag-CRY1
C
N+C
N
Supplemental Fig.8 CRY1 did not alter PER2 subcellular localization. (A) Subcellular localizations of either PER2-WT, PER2-NLS or PER2-NES were quantified; N, nucleus; C, cytoplasm; N+C, both nucleus and cytoplasm. (B) Subcellular localizations of either PER2-WT, PER2-NLS or PER2-NES were quantified when CRY1 was co-expressed. The data represented are the mean ± SEM of three independent samples. *p<0.05, **p<0.01, ***p<0.001, compared to each subcellular localization in cells expressing PER2-WT by Student’s two-tailed t test.

## Slide 11
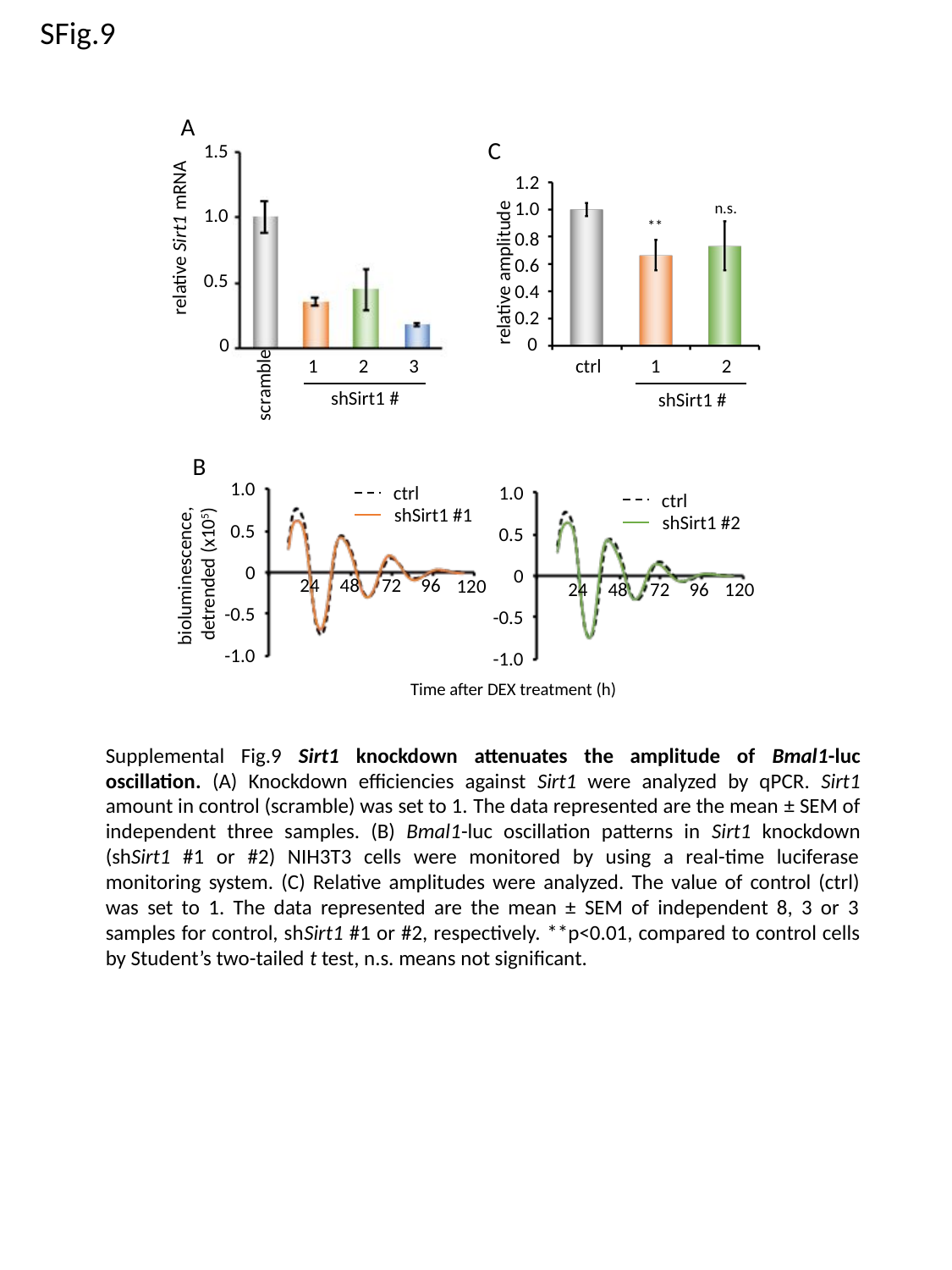

SFig.9
A
C
1.5
1.0
relative Sirt1 mRNA
0.5
0
1
2
3
scramble
shSirt1 #
1.2
1.0
0.8
0.6
relative amplitude
0.4
0.2
0
ctrl
1
2
shSirt1 #
n.s.
**
B
1.0
ctrl
shSirt1 #1
0.5
0
24
48
72
96
120
-0.5
-1.0
bioluminescence,
 detrended (x105)
1.0
ctrl
shSirt1 #2
0.5
0
24
48
72
96
120
-0.5
-1.0
Time after DEX treatment (h)
Supplemental Fig.9 Sirt1 knockdown attenuates the amplitude of Bmal1-luc oscillation. (A) Knockdown efficiencies against Sirt1 were analyzed by qPCR. Sirt1 amount in control (scramble) was set to 1. The data represented are the mean ± SEM of independent three samples. (B) Bmal1-luc oscillation patterns in Sirt1 knockdown (shSirt1 #1 or #2) NIH3T3 cells were monitored by using a real-time luciferase monitoring system. (C) Relative amplitudes were analyzed. The value of control (ctrl) was set to 1. The data represented are the mean ± SEM of independent 8, 3 or 3 samples for control, shSirt1 #1 or #2, respectively. **p<0.01, compared to control cells by Student’s two-tailed t test, n.s. means not significant.
